# Supplementary material for: Generation of canine induced pluripotent stem cells under feeder-free conditions using Sendai virus vector encoding six canine reprogramming factors
Source: Stem Cell Reports. 2023 Dec 21;19(1):141–57. doi: 10.1016/j.stemcr.2023.11.010 (PMC10828825; doi:10.1016/j.stemcr.2023.11.010)
Supplement: Document S1. Figures S1–S6, Tables S1–S3, and supplemental experimental procedures [file mmc1.pdf]

Stem Cell Reports, Volume *19*

Supplemental Information

Generation of canine induced pluripotent stem cells under feeder-free conditions using Sendai virus vector encoding six canine reprogramming factors

Masaya Tsukamoto, Kazuto Kimura, Takumi Yoshida, Miyuu Tanaka, Mitsuru Kuwamura, Taro Ayabe, Genki Ishihara, Kei Watanabe, Mika Okada, Minoru Iijima, Mahito Nakanishi, Hidenori Akutsu, Kikuya Sugiura, and Shingo Hatoya

Figure S1, Canine sequence and canine 6-factors SeV

A

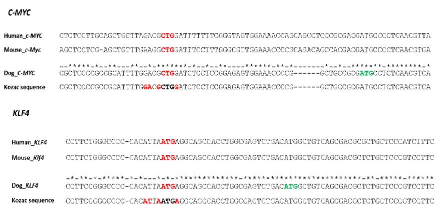

B

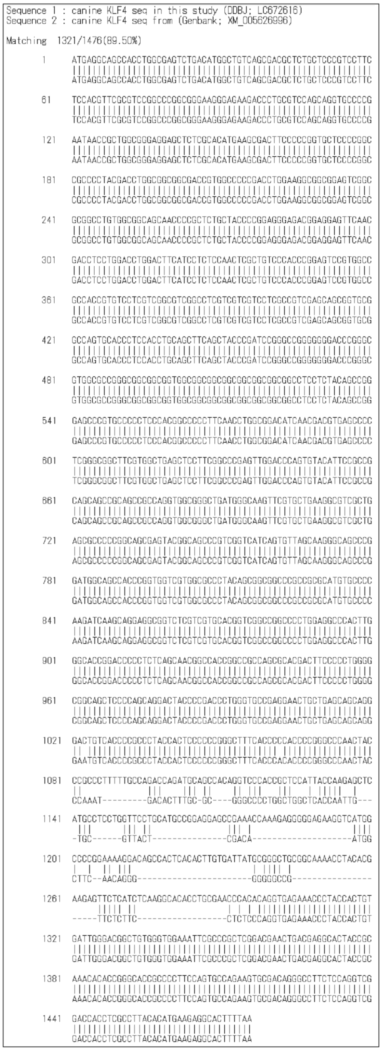

C

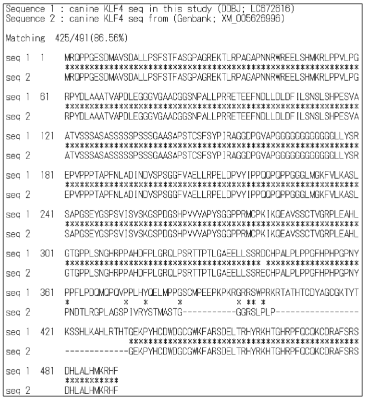

D

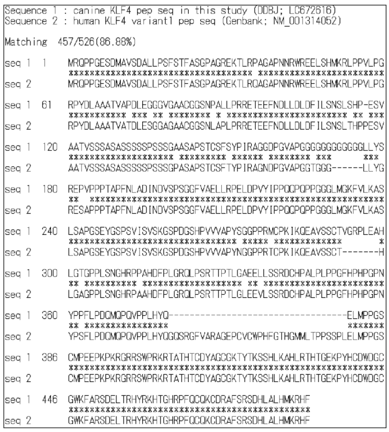

E

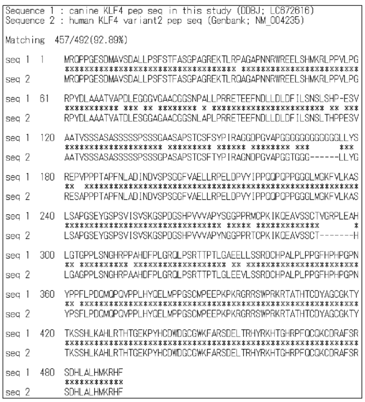

F

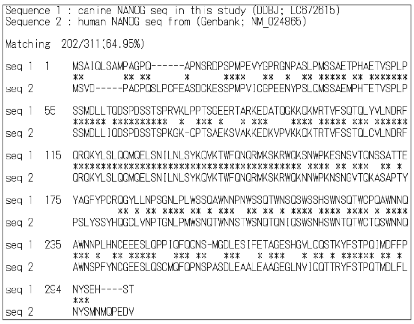

G

Gene	Canine	Human	Homology (%)
LIN28A	XM_849892.5	NM_024674.6	94.76
OCT3/4	XM_538830.4	NM_002701.6	90.83
SOX2	XM_005639752.4	NM_003106.4	98.14
C-MYC	NM_001003246.2	NM_002467.6	92.29
KLF4	LC672616	NM_004235.6	92.89
NANOG	LC672615	NM_024865.4	64.95

H

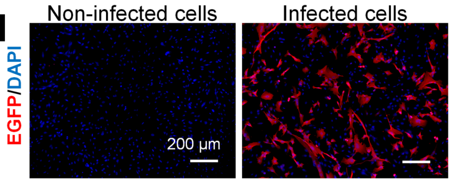

I

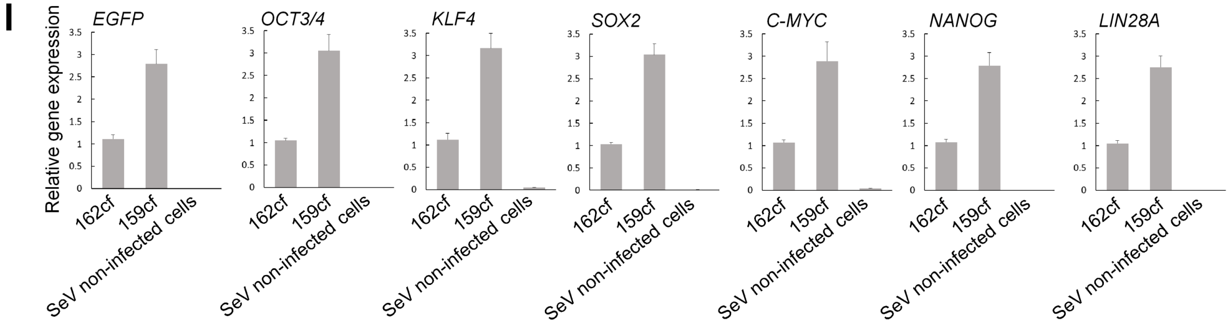

Figure S2, Reprogramming CEFs with feeder cells

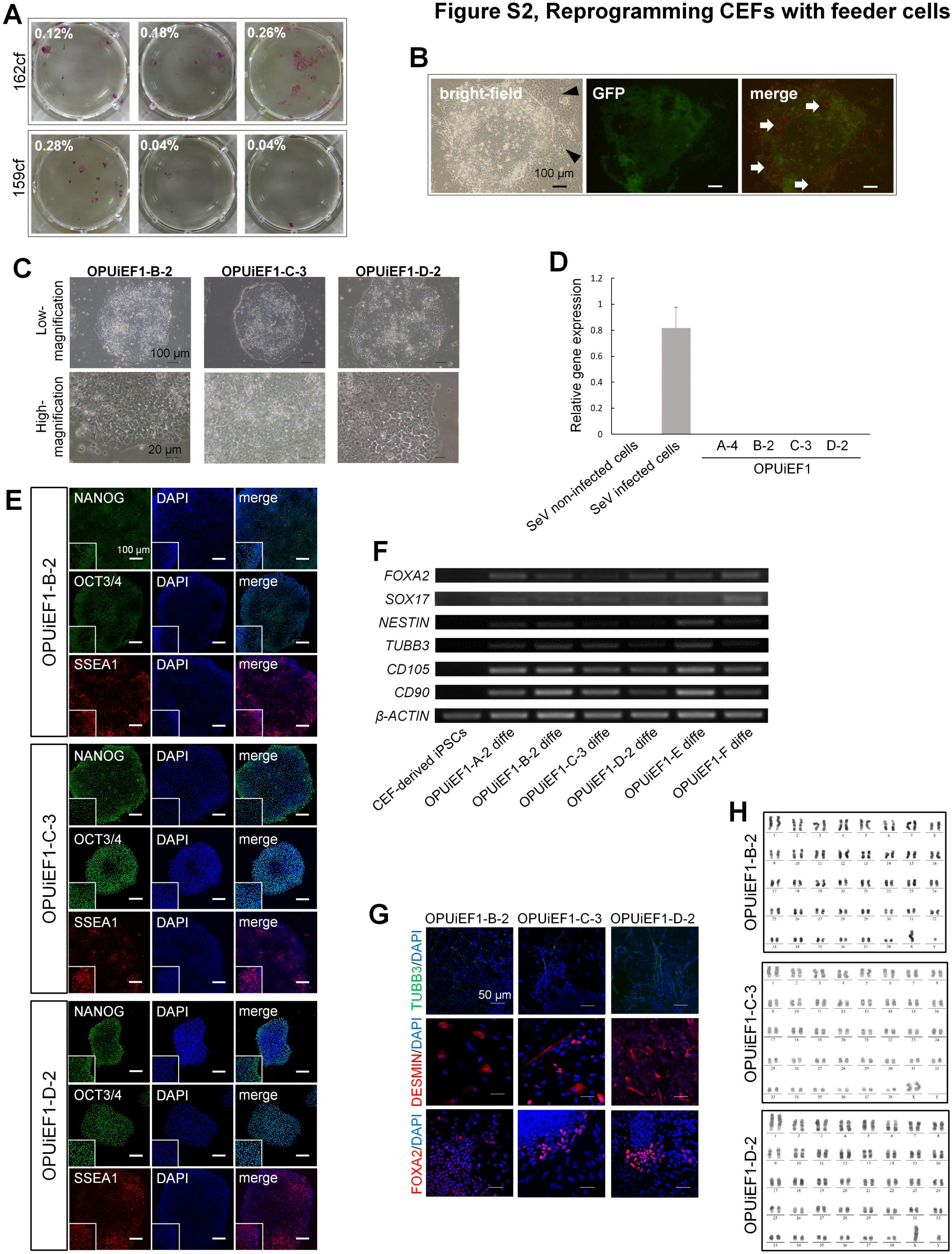

Figure S3, Reprogramming CDFs with feeder cells

A

	159cf	162cf
#1 (n = 2)	0.0393%	0
#2 (n = 1)	0.00870%	0.00870%

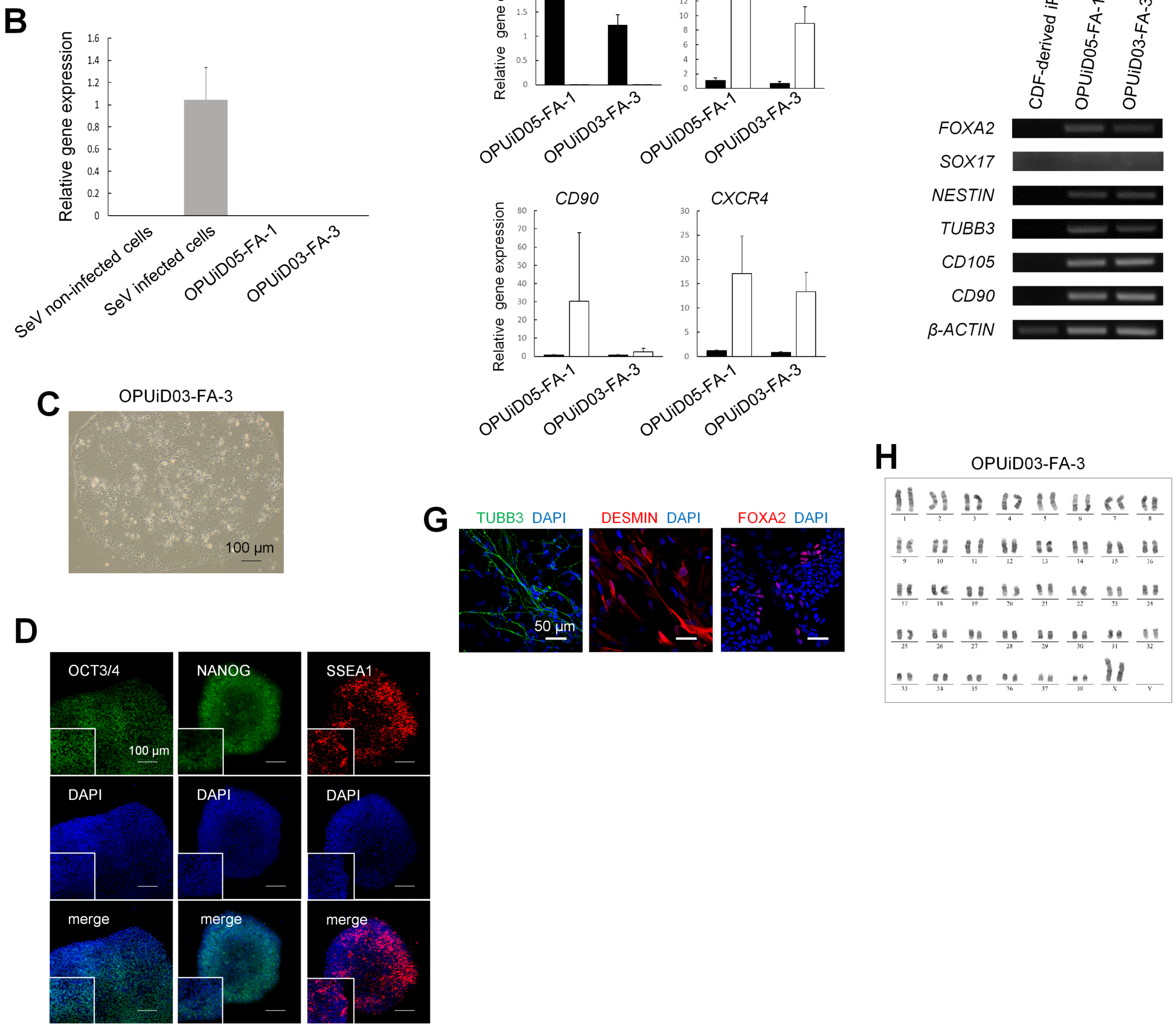

Figure S4, Comparison of cUC derivation method

A

Cell number at day 10

individuals	isolation method: conventional	isolation method: combination
No. 1	N/D	3.8×10^5
No. 2	N/D	1.7×10^5
No. 3	2.2×10^3	4.8×10^4
No. 4	1.9×10^4	3.4×10^4
No. 5	2.1×10^4	7.9×10^4

B

Doubling time (days)

individuals	isolation method: conventional	isolation method: combination
No. 1	N/D	0.51
No. 2	N/D	0.99
No. 3	*1	0.83
No. 4	2.91	0.55
No. 5	*1	1.60

C

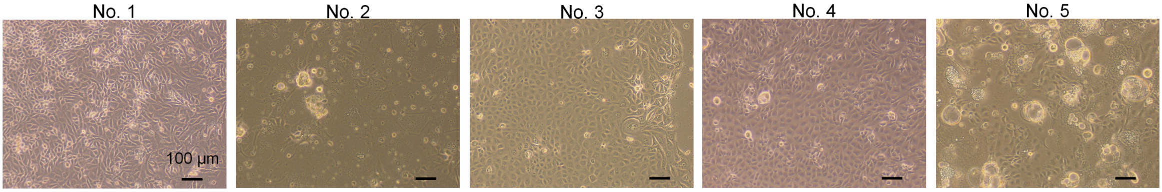

D

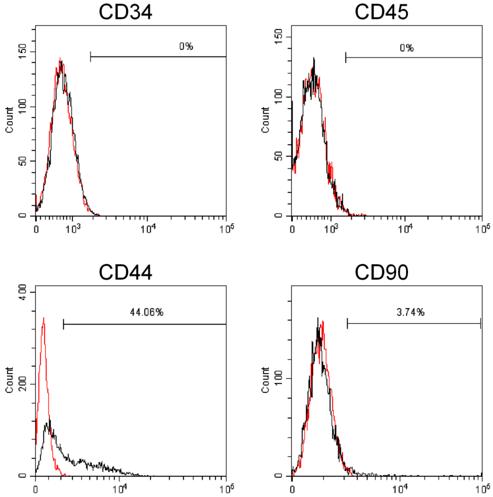

E

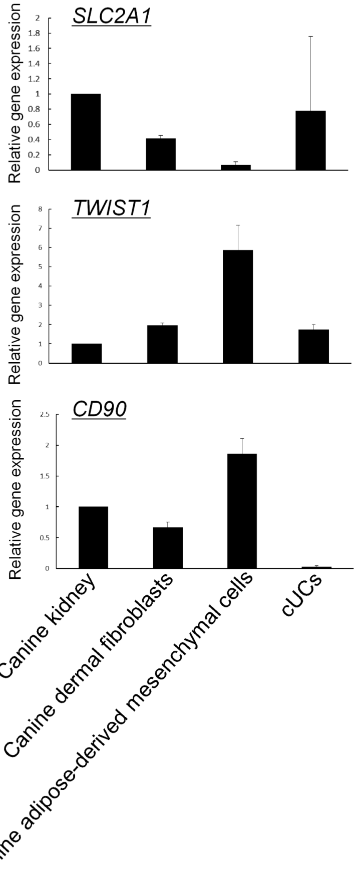

Figure S5, Reprogramming cUCs with feeder cells

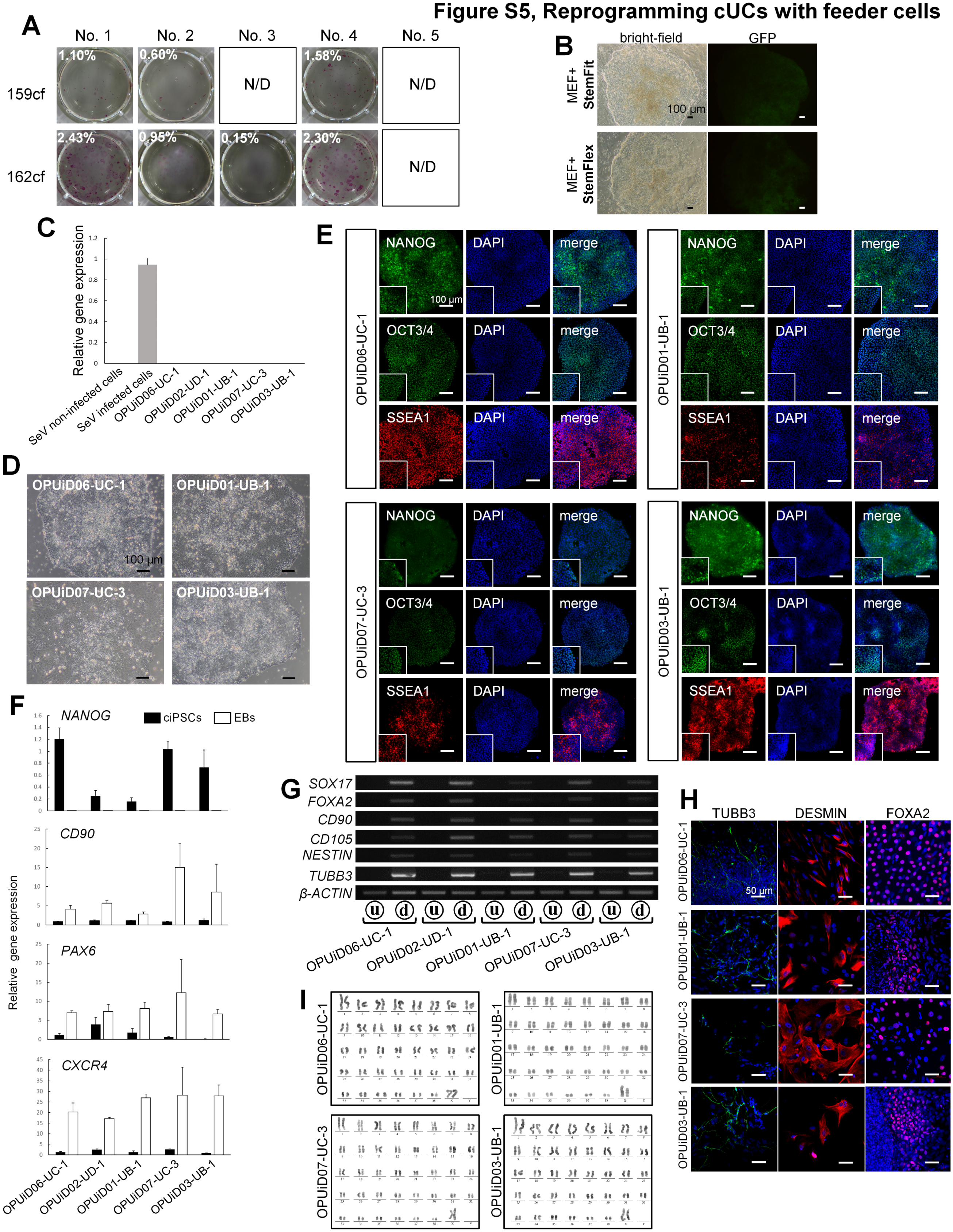

Figure S6, ciPSC generated without feeder cells

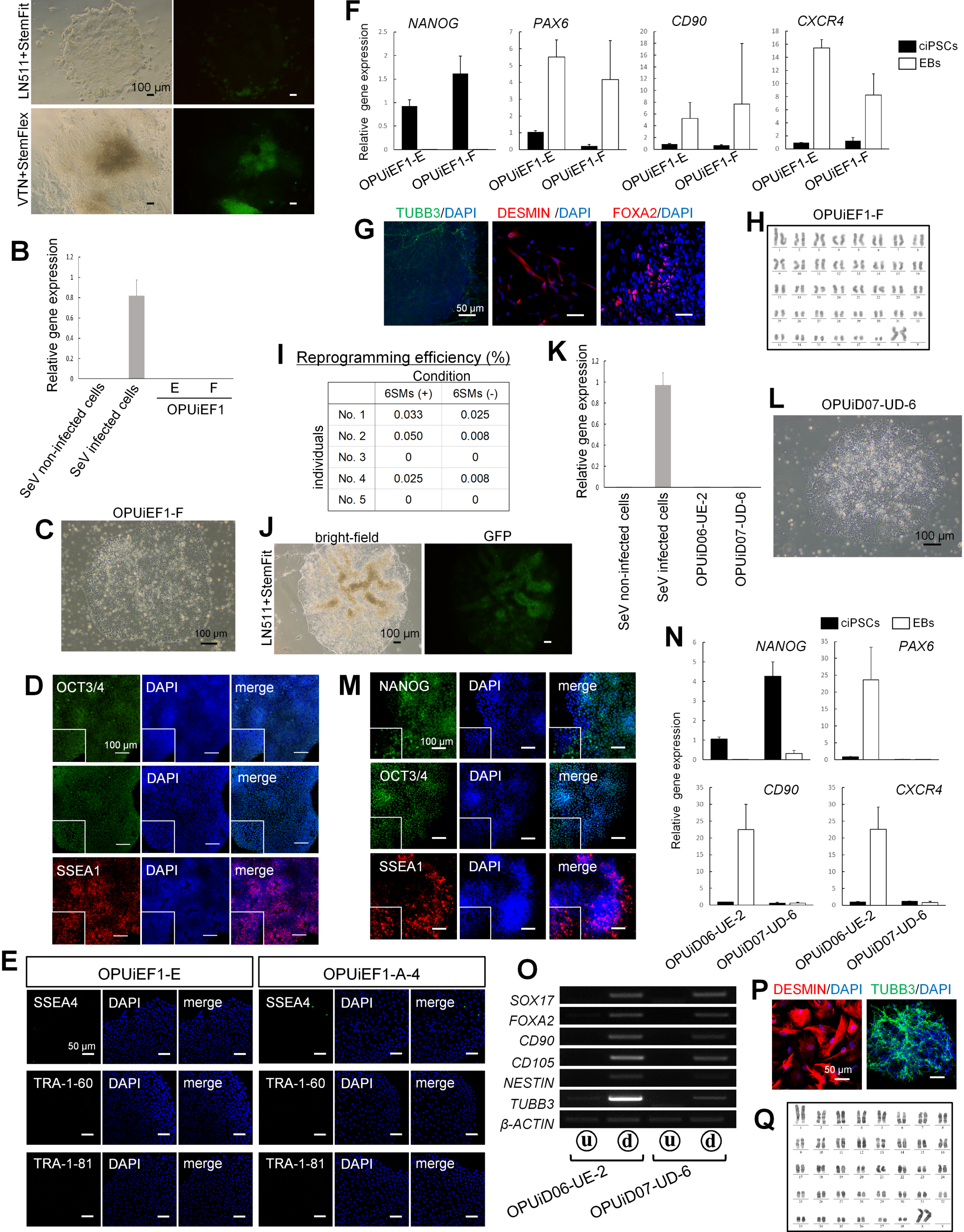

Reprogramming efficiency (%)

individuals	Condition 6SMs (+)	Condition 6SMs (-)
No. 1	0.033	0.025
No. 2	0.050	0.008
No. 3	0	0
No. 4	0.025	0.008
No. 5	0	0

Supplemental Figure Legends

Figure S1 (related to Figure 1) Comparison of pluripotency associated genes and function of canine 6-factors Sendai virus (SeV)
(A) Comparison of *C-MYC* and *KLF4* sequences from GenBank. Bases in red font in human and mouse genes represent start codons. Bases in green font in dog *C-MYC* and *KLF4* are start codons from predicted data registered in GenBank. The start codon determined in this study is shown in red font and is 39 bases upstream of *C-MYC* and 27 bases upstream of *KLF4*. These start codon locations are identical to those of human and mouse genes. Kozak sequences are presented at the bottom.
(B) Sequencing results for *KLF4* (canine *KLF4* seq in this study) and sequence comparison with the sequence from XM_005626996 (canine *KLF4* seq from GenBank). The homology is 89.50%.
(C) Amino acid homology between KLF4 sequence determined in this study (canine KLF4 pep seq in this study) and canine KLF4 sequence from GenBank (canine KLF4 pep seq from GenBank). The homology is 86.56%.
(D) Comparison between canine KLF4 determined in this study and human KLF4 splicing variant 1. The homology is 86.88%.
(E) Comparison between canine KLF4 determined in this study and human KLF4 splicing variant 2. The homology is 92.89%.
(F) Comparison between canine NANOG determined in this study and human NANOG. The homology is 64.95%.
(G) Summary of homology for six reprogramming genes between canine and human.
(H) Immunostaining of enhanced green fluorescence protein (EGFP). Infection efficiency was calculated from EGFP-positive cells and total cells. Infection efficiency was 33.7% (n=3).
(I) qPCR analysis of canine pluripotency-associated genes and *EGFP* in 162cf or 159cf-infected canine embryonic fibroblasts (CEFs) and Sendai virus (SeV) non-infected cells. *β-ACTIN* was used as a normalization control gene. The relative gene expression levels were determined relative to expression in 162cf-infected cells.

Figure S2 (related to Figure 1 and 2) Reprogramming of CEFs using canine six-factors SeV.
(A) Alkaline phosphatase staining of primary colonies. The reprogramming efficiency of each event is shown.
(B) Images obtained after one small interfering RNA (siRNA) procedure. The black arrowhead shows differentiated cells, and the white arrow shows EGFP-negative undifferentiated cells. Scale bar = 100 μm.
(C) Morphologies of each ciPSCs, OPUiEF1-B-2, -C-3, and -D-2. Scale bar = 100 or 20 μm.
(D) qPCR for SeV vector in four CEF-derived iPSCs. SeV non-infected cells and SeV-infected cells were used as negative and positive control, respectively. *β-ACTIN* was used as a normalization control gene.
(E) Immunocytochemistry of OPUiEF1-B-2, OPUiEF1-C-3, and OPUiEF1-D-2 for pluripotent markers OCT3/4, NANOG, and SSEA1. Scale bar = 100 μm. High-magnification images are shown as insets.
(F) RT-PCR of differentiation markers after spontaneous differentiation of CEF-derived iPSCs under two-dimensional conditions. Endodermal markers *FOXA2* and *SOX17*, ectodermal markers *NESTIN* and *TUBB3*, and mesodermal markers *CD105* and *CD90*. *β-ACTIN* was used as a normalization control gene; CEF-derived iPSCs were used as negative controls.
(G) Immunocytochemistry of each differentiation marker after spontaneous differentiation. Ectodermal marker TUBB3, mesodermal marker DESMIN, and endodermal markers FOXA2. Scale bar = 50 μm.
(H) Karyotype analysis of each CEF-derived iPSCs, OPUiEF1-B-2, OPUiEF1-C-3, and OPUiEF1-D-2.

Figure S3 (related to Figure 3) Reprogramming of canine dermal fibroblasts (CDFs) using canine six-factors SeV and characteristics of CDF-derived iPSCs
(A) Reprogramming efficiency of CDFs from two individuals.
(B) qPCR of SeV vector for two CDF-derived iPSCs. SeV non-infected cells and SeV infected cells were used as negative and positive control, respectively. *β-ACTIN* was used as a

normalization control gene.
(C) Morphologies of OPUiD03-FA-3. Scale bar = 100 μm.
(D) Immunocytochemistry of OPUiD03-FA-3 for pluripotent markers OCT3/4, NANOG, and SSEA1. Scale bar = 100 μm.
(E) qPCR analysis of undifferentiated and differentiation markers 12 days after embryoid body (EB) formation. Black bar and white bar represent ciPSCs and EBs, respectively. Undifferentiated marker *NANOG*, ectodermal marker *PAX6*, mesodermal marker *CD90*, and endodermal marker *CXCR4*. *β-ACTIN* was used as a normalization control gene, and data are shown as the mean ± standard deviation (n = 3).
(F) RT-PCR for differentiation markers after spontaneous differentiation of CDF-derived iPSCs under two-dimensional conditions. Endodermal markers *FOXA2* and *SOX17*, ectodermal markers *NESTIN* and *TUBB3*, and mesodermal markers *CD105* and *CD90*. *β-ACTIN* was used as a normalization control gene; CDF-derived iPSCs were used as negative controls.
(G) Immunocytochemistry of each differentiation marker of OPUiD03-FA-3 after spontaneous differentiation via EB formation. Ectodermal marker TUBB3, mesodermal marker DESMIN, and endodermal markers FOXA2. Scale bar = 50 μm.
(H) Karyotype analysis of OPUiD03-FA-3 at passage 15.

Figure S4 (related to Figure 4) Comparison of two isolation methods of canine urine-derived cells (cUCs).
(A) Cell number at day10 between two isolation methods. N/D in dog No. 1 and No. 2 means not performed because urine sample was not enough to divide into two groups.
(B) Doubling time of cUCs isolated using each isolation method. N/D in dog No. 1 and No. 2 means not performed. *1 in dog No. 3 and No. 5 means that cell growth was not observed after culture.
(C) Morphologies of cUCs from each individual. Scale bar = 100 μm.
(D) Flow cytometry analysis of cUCs for CD34, CD44, CD45, and CD90. Isotype control for each marker was used as negative control and is indicated by a red line.
(E) qPCR for *SLC2A1* (renal epithelial marker), *TWIST1* and *CD90* (mesenchymal marker). Expression levels in each sample were standardized to the level of *β-ACTIN*. The data are presented as the mean ± standard deviation (n = 4), except for canine kidney data, which represents a single repricate and is included as supplemental reference data.

Figure S5 (related to Figure 4) Reprogramming of cUCs and characteristics of cUC-derived iPSCs.
(A) Alkaline phosphatase staining of primary colonies obtained from each condition. N/D in dogs No. 3 and No. 5 indicates no observation of primary colonies. The right table shows the reprogramming efficiencies. Each reprograming experiment was performed one time.
(B) Primary colony morphologies generated under StemFit and StemFlex. Scale bar = 100 μm.
(C) qPCR of SeV vector for five cUC-derived iPSCs. SeV non-infected cells and SeV infected cells were used as negative and positive control, respectively. *β-ACTIN* was used as a normalization control gene.
(D) Morphologies of ciPSCs, OPUiD06-UC-1, OPUiD01-UB-1, OPUiD07-UC-3, and OPUiD03-UB-1. Scale bar = 100 μm.
(E) Immunocytochemistry of cUC-derived iPSCs for pluripotent markers OCT3/4, NANOG, and SSEA1. Scale bar = 100 μm. High-magnification images are shown as insets.
(F) qPCR analysis of undifferentiated and differentiation markers at 12 days after EB formation. Black bar and white bar represent ciPSCs and EBs, respectively. Undifferentiated marker *NANOG*, ectodermal marker *PAX6*, mesodermal marker *CD90*, and endodermal marker *CXCR4*. *β-ACTIN* was used as a normalization control gene, and data are shown as the mean ± standard deviation (n = 3).
(G) RT-PCR of differentiation markers after spontaneous differentiation of cUC-derived iPSCs under two-dimensional conditions. Endodermal markers *FOXA2* and *SOX17*, ectodermal markers *NESTIN* and *TUBB3*, and mesodermal markers *CD105* and *CD90*. *β-ACTIN* was used as a normalization control gene; cUC-derived iPSCs were used as negative controls (u). The left right lanes of each ciPSC line show the cells after differentiation (d).
(H) EB formation from cUC-derived iPSCs and immunocytochemistry of EBs for each differentiation marker. Ectodermal marker TUBB3, mesodermal marker DESMIN, and

endodermal markers FOXA2. Scale bar = 50 μm.
(I) Karyotype analysis of OPUiD06-UC-1, OPUiD01-UB-1, OPUiD07-UC-3, and OPUiD03-UB-1 at passage 15, 11, 15, and 15, respectively.

Figure S6 (related to Figure 5 and 6) Characteristics of iPSCs generated under feeder-free conditions.
(A) Morphologies of primary colonies obtained from CEFs under combinations of iMatrix-511 (LN511) with StemFit or Vitronectin (VTN) with StemFlex. Scale bar = 100 μm.
(B) qPCR of SeV vector for OPUiEF1-E and -F. SeV non-infected cells and SeV-infected cells were used as negative and positive control, respectively. *β-ACTIN* was used as a normalization control gene.
(C) Morphologies of OPUiEF1-F. Scale bar = 100 μm.
(D) Immunocytochemistry of OPUiEF-F for pluripotent markers OCT3/4, NANOG, and SSEA1. Scale bar = 100 μm. High-magnification images are shown as insets.
(E) Immunocytochemistry of CEF-derived iPSCs under feeder-free conditions (OPUiEF1-E) and with feeder cells (OPUiEF1-A-4) for SSEA4, TRA-1-60, and TRA-1-81. Scale bar = 50 μm. Both ciPSCs did not express these undifferentiated markers.
(F) qPCR analysis of undifferentiated and differentiation markers 12 days after EB formation from CEF-derived iPSCs. Black bar and white bar represent ciPSCs and EBs, respectively. Undifferentiated marker *NANOG*, ectodermal marker *PAX6*, mesodermal marker *CD90*, and endodermal marker *CXCR4*. *β-ACTIN* was used as a normalization control gene, and data are shown as the mean ± standard deviation (n = 3).
(G) Immunocytochemistry for each differentiation marker of OPUiEF1-F after spontaneous differentiation under two-dimensional conditions. Ectodermal marker TUBB3, mesodermal marker DESMIN, and endodermal markers FOXA2. Scale bar = 50 μm.
(H) Karyotype analysis of OPUiEF1-F at passage 28.
(I) Reprogramming efficiencies of cUCs. Each reprograming experiment was performed one time.
(J) Primary colony morphologies generated from cUCs under the combination of iMatrix-511 (LN511) and StemFit. Scale bar = 100 μm.
(K) qPCR of SeV vector in both ciPSCs, OPUiD06-UE-2 and OPUiD07-UD-6. SeV non-infected cells and SeV-infected cells were used as negative and positive control, respectively. *β-ACTIN* was used as a normalization control gene.
(L) Morphologies of ciPSCs, OPUiD07-UD-6. Scale bar = 100 μm.
(M) Immunocytochemistry of OPUiD07-UD-6 for undifferentiated markers. Scale bar = 100 μm.
(N) qPCR analysis of undifferentiated and differentiation markers at 12 days after EB formation from cUC-derived iPSCs. Black bar and white bar represent ciPSCs and EBs, respectively. Undifferentiated marker *NANOG*, ectodermal marker *PAX6*, mesodermal marker *CD90*, and endodermal marker *CXCR4*. *β-ACTIN* was used as a normalization control gene, and data are shown as the mean ± standard deviation (n = 3). OPUiD07-UD-6 did not express differentiation markers.
(O) RT-PCR of differentiation markers after spontaneous differentiation of OPUiD06-UE-2 and OPUiD07-UD-6 under two-dimensional conditions. Endodermal markers *FOXA2* and *SOX17*, ectodermal markers *NESTIN* and *TUBB3*, and mesodermal markers *CD105* and *CD90*. *β-ACTIN* was used as a normalization control gene; iPSCs before differentiation were used as negative controls (u). Right lanes of each ciPSC line show the cells after differentiation (d).
(P) Immunocytochemistry of each differentiation marker of OPUD07-UD-6 after spontaneous differentiation via EB formation. Only mesodermal marker DESMIN and ectodermal marker TUBB3 were detected. Scale bar = 50 μm.
(Q) Karyotype analysis of OPUiD07-UD-6 at passage 19.

Table S1, Summary information on passage numbers

cell line name (OPUi)	RT-PCR for SeV	qPCR for SeV	IF (pluripotency)	qPCR (pluripotency)	IF (differentiation)	qPCR (differentiation)	RT-PCR (differentiation)	teratoma assay	karyotyping	cell source	feeder cell
EF1-A-4	5	12-14	15	12-14	10	13-16	28	10	14	CEF	yes
EF1-B-2	4	13-15	16	13-15	12	14-16	38	10	15		
EF1-C-3	12	14-17	18	14-17	27	18-20	34	17	20		
EF1-D-2	6	21-23	12	21-23	12	21-23	43	16	26		
EF1-E	11	19-21	22	19-21	19	19-21	48	21	17	CEF	no
EF1-F	11	15-17	13	15-17	21	18-20	47	30	28		
D05-FA-1	5	14-16	13	14-16	12	14-16	22	11	15	CDF	yes
D03-FA-3	3	11-13	11	11-13	9	12-14	18	8	15		
D06-UC-1	3	10-12	12	10-12	12	10-12	26	10	15	cUC	yes
D02-UD-1	4	10-12	14	10-12	14	10-12	25	12	15		
D01-UB-1	4	10-12	14	10-12	17	10-12	28	12	11		
D07-UC-3	4	15-17	13	15-17	14	15-17	23	11	15		
D03-UB-1	5	34-36	16	34-36	20	33-35	19	11	15		
D06-UE-2	11	17-19	21	17-19	20	17-19	11	12	25	cUC	no
D07-UD-6	5	10-12	17	10-12	16	10-12	9	11	19		

IF; immunofluorescence, SeV; Sendai virus, CEF; canine embryonic fibroblast, CDF; canine dermal fibroblast, cUC; canine urine derived cell

Table S2, Oligo sequence

Sequence of siRNA for SeV

	Sequence (5' → 3')
Sense	CAAUAGUUCACGCUGAAAGUG
Anti-sense	CUUUCAGCGUGAACUAUUGCU

Sequence of primers

Name		Sequence (5' → 3')
β-ACTIN	Forw ard	CAGCAAATGTGGATCAGC
	Reverse	CCAATCTCATCTCGGTTTC
OCT3/4	Forw ard	CTGAAGCAGAAGAGGATCAC
	Reverse	GCCGCAGCTTACACATATTC
NANOG	Forw ard	CTAGGGACCCTTCTCCAATG
	Reverse	CTTCTGTTTCTTGCCCTGG
SOX2	Forw ard	AACCCCAAGATGCACAACTC
	Reverse	CGGGGCCGGTATTTATAATC
KLF4	Forw ard	ACACTTGTGATTATGCGGGC
	Reverse	CCTCGTCAGTTCGTCCGAG
C-MYC	Forw ard	TCCTCCGGAGAGTGGAAACC
	Reverse	CCGAGTCGTAGTCGAGGTCA
LIN28A	Forw ard	AGAACATGCAGAAGCGCAGA
	Reverse	TTGCATTCCTTGGCATGGTG
EGFP	Forw ard	GGCACAAGCTGGAGTACAAC
	Reverse	ATGCCGTTCTTCTGCTTGTC
NESTIN	Forw ard	GTTCCAGAGGCTATACAGG
	Reverse	CTAGAGTGATAAGGGCTGAG
TUBB3	Forw ard	CCGGAACCATGGACAGCGTC
	Reverse	AGCGGAGAGAAGTAGTGACG
CD90	Forw ard	ACCTGGAGGAGGAAGAGGAA
	Reverse	AAAGCTCCTGGAAATGCTCA
CD105	Forw ard	ACCGTGGAACTCATCCAGAG
	Reverse	AGGTAAGGGTGCCAGTTGTG
FOXA2	Forw ard	CAAGGCCTATGAACAGGTG
	Reverse	CTGGTAGTAGGAGGTGTCTGC
SOX17	Forw ard	AAGATGCTGGGCAAGTCG
	Reverse	CCCTCCACCCGCTTCAG
SeV	Forw ard	GGAAGGAATCGGCTCAGTGATG
	Reverse	GGGCCGTGTTCATGGTCAC
SLC2A1	Forw ard	GATCGGCTCCTTCTCTGTGG
	Reverse	AGGACTTGGCCAGTTTCGAG
TWIST1	Forw ard	AGCTACGCCTTCTCGGTCTG
	Reverse	CTGTCCATTTTCTCCTTCTCTGG
CDH2	Forw ard	AGCACCCTCCTCAGTCAACG
	Reverse	TGTCAACATGGTCCCAGCA
ZEB1	Forw ard	GTGTGGGGTGTGAGAACTTGA
	Reverse	TCTGGTTCTCTTGAAAGGTTAGG
EPCAM	Forw ard	TCGCTGTCATTGTGGTTGTG
	Reverse	TGCATCTCACCCATCTCCTTT
CDH1	Forw ard	TCCTGGGCAGGGTGAGTT
	Reverse	GAGGCCGCTTGACTGTAATC
UTF1	Forw ard	GCTGCTGACCCTGAACCAG
	Reverse	CCCAAGATGAAGCCCACGG
ESRRB	Forw ard	GGAGGCGTGCTAGAGATGAA
	Reverse	GGACTGGTCACCACTAAGGG
GATA4	Forw ard	ACCAGCAGCAGTGAAGAGATG
	Reverse	AGATGTGTAGCCTTGTGGGG
GATA6	Forw ard	CACTACTTGTGCAACGCCTG
	Reverse	CACAAGACAATCCAAGCCGC
PAX6	Forw ard	TGCTGGACAATCAAAACGTGTC
	Reverse	TGGAGTCGCTACTCTCGGTT
CXCR4	Forw ard	GTTGAGGCTGTGGCAAACTG
	Reverse	GTAGACCACCTTTTCCGCCA

Table S3, Antibody

Primary antibodies for immunocytochemistry

Antigen	Cat.no.	Source	Final concentration
OCT3/4	sc-5279	Santa Cruz Biotechnology	0.4 μg/mL
NANOG	ab77095	Abcam	5 μg/mL
SSEA1	MAB4301	Millipore	1:100
TUBB3	MAB1637	Millipore	1:400
DESMIN	ab82506	Abcam	1 μg/mL
FOXA2	720061	Thermo Fisher Scientific	2.5 μg/mL
GFP	A-6455	Thermo Fisher Scientific	1:1000

Secondary antibodies for immunocytochemistry

	Cat.no.	Source	Final concentration
Goat anti-Mouse IgG, Alexa Fluor 488	A11029	Invitrogen	1 μg/mL
Goat anti-Rabbit IgG, Alexa Fluor 546	A11010	Invitrogen	4 μg/mL
Rabbit anti-Goat IgG, Alexa Fluor 488	A27012	Invitrogen	1 μg/mL
Goat anti-Mouse IgM, Cy3	AP128C	Chemicon	1 μg/mL

Antibodies for flow cytometry

Antigen	Cat.no.	Source	Final concentration
CD34	12-0340-42	Thermo Fisher Scientific	2.5 μg/mL
CD44	11-5440-42	Thermo Fisher Scientific	1.25 μg/mL
CD45	11-5450-42	Thermo Fisher Scientific	2.5 μg/mL
CD90	12-5900-42	Thermo Fisher Scientific	5 μg/mL

Supplemental experimental procedures

Sequencing of canine pluripotency-associated genes

NANOG was resequenced using ENSCAFT00000022072.3 (Ensembl 94) as the genomic DNA template; each exon was resequenced. *KLF4* was resequenced using cDNA from the canine ovary as a template. KOD FX DNA polymerase (Toyobo, Osaka, Japan) and a Takara Dice Touch thermal cycler (Shiga, Japan) were used for PCR. Sequencing was outsourced to Eurofins Genetics (Tokyo, Japan).

The 5′-Full RACE Core Set (Takara) was used for 5′ rapid amplification of cDNA ends (RACE) to identify the start codon of canine *KLF4* and *C-MYC* using reverse transcribed cDNA from the canine ovary as a template. The gene-specific phosphorylated primer sequences used in the 5' RACE were KLF4: TCAGCAGTTCCTC-P and C-MYC: TGATCTGTTTCAGG-P. P were used. The sequences of the primers used for 1st and 2nd PCR were KLF4 (1st-Forward: GGAATGTACACTGGGTCCAACT, 1st-Reverse: GTCATCAGTGTTAGCAAGGG, 2nd-Forward: AGTTGGAGAGGATGAAGTCCAG, 2nd-Reverse: CAGCTCCCCAGCAGGACTAC), C-MYC (1st-Forward: TTTCTTCCAGATATCCTCGCTG, 1st-Reverse: TCAAAAACATCATCATCCAGGA, 2nd-Forward: CTGCTGGTAGAAGTTCTCCTCC, 2nd-Reverse: GGAAGAACAAGGACGAAGAA).

Construction of canine six factors-SeV

Canine six factors-SeV was constructed according to the method described in the patent[1]. Briefly, full-length genome cDNA installed with the six canine pluripotency-associated genes (Oct3/4, Klf2, Sox2, c-Myc, Nanog and Lin28), the codon-optimized Sendai virus NP, P, C and L genes, the enhanced green fluorescent protein (EGFP) gene and puromycin N-acetyl transferase (PuroR) gene was constructed on a low-copy plasmid vector with P15A replication origin. This cDNA and the plasmid vectors for expressing Sendai virus NP, P and L proteins were transfected to BHK-21 cells expressing T7 RNA polymerase for reconstituting active nucleocapsid in the cell. Finally, the plasmid vectors for expressing Sendai virus F, HN and M proteins were transfected to the BHK-21 cells containing the vector genome for producing Canine six factors-SeV. SeV 159cf vector and SeV 162cf vector have different gene expression levels. 159cf expresses the installed genes about three times higher than 162cf does, by controlling NP gene expression[1].

Composition of culture media

Feeder medium is composed of Dulbecco’s modified Eagle’s medium with high glucose (DMEM high-glucose; Nacalai Tesque, Kyoto, Japan) containing 10% fetal bovine serum (FBS; Kibbutz Beik Haemek, Israel), 2 mM L-glutamine (Nacalai Tesque), 100 U/mL penicillin, and 100 µg/mL streptomycin (Nacalai Tesque). The primary medium was composed of DMEM high glucose and DMEM/Nutrient Mixture F-12 Ham (Nacalai Tesque) mix (1:1) supplemented with 10% FBS, 100 U/mL penicillin, 100 µg/mL streptomycin, 2.5 µg/mL amphotericin B, and REGM SingleQuot kit supplements (Lonza, Basel, Switzerland). RE/MC proliferation medium was mixed RE proliferation medium and MC proliferation medium (1:1). RE proliferation medium is RE cell basal medium containing REGM Bullet Kit (Lonza). MC proliferation medium was DMEM high-glucose supplemented with 10% FBS, 100 U/mL penicillin, 100 µg/mL streptomycin, GlutaMAX (1×, Thermo Fisher Scientific, Waltham, MA, USA), 0.1 mM minimal essential medium nonessential amino acids (MEM NEAA; Thermo Fisher Scientific), 5 ng/mL basic fibroblast growth factor (Thermo Fisher Scientific), 5 ng/mL platelet derived growth factor-AB (Peprotech, Rocky Hill, NJ, USA), and 5 ng/mL epidermal growth factor (Thermo Fisher Scientific). N2B27 medium consisted of DMEM/Nutrient Mixture F-12 Ham supplemented with N2 supplement (1×, Thermo Fisher Scientific), B27 supplement (1×, Thermo Fisher Scientific), GlutaMAX, 0.1 mM MEM NEAA, and 0.1 mM 2-mercaptoethanol (Sigma Aldrich, St. Louis, MO, USA). The 20% FBS medium consisted of DMEM/Nutrient Mixture F-12 Ham supplemented with 20% FBS (Sigma Aldrich), 2mM L-glutamine, 100 U/mL penicillin, 100 µg/mL streptomycin, 0.1 mM MEM NEAA, and 0.1 mM 2-mercaptoethanol. The small molecule cocktails added to N2B27 medium were composed of 10 µM Y-27632 (Nacalai Tesque), 0.5 µM PD0325901 (Reprocell, Kanagawa, Japan), 3 µM CHIR99021 (Fujifilm Wako Pure Chemical Corporation, Osaka, Japan), 0.5 µM A83-01 (Stemgent, San Diego, CA, USA), named “4SMs,” or 10 µM forskolin (Tocris, Bristol, Avon, UK), and 50 µg/mL L-ascorbic acid (Sigma Aldrich) in addition to 4SMs, named as “6SMs.”

Preparation and culture of feeder cells and canine cells

MEFs were isolated from the fetuses of ICR mice (Japan SLC, Shizuoka, Japan). CEFs were isolated from the intrauterine fetus of a beagle dog after 30 days of pregnancy. The head, visceral tissues, and liver were removed from the fetus. The remaining tissues were cut into small pieces and cultured in a feeder medium. CDFs were derived from punch biopsy tissue taken from the dorsal skin of 8- and 12-year-old beagle bitches. After chopping, the tissues were cultured in a feeder medium.

cUCs were isolated from urine samples from five dogs (from 9 to 14 years old bitches). Urine samples were obtained with a sterile catheter. cUCs were isolated as described previously[2]. Briefly, urine samples were centrifuged at 400 × g for 10 min, and the supernatant was removed. The pellet was resuspended with washing buffer, which was PBS (-) containing 100 U/mL penicillin, 100 µg/mL streptomycin, and 500 ng/mL amphotericin B (Sigma Aldrich), and centrifuged at 200 × *g* for 10 min. After removing the supernatant, for the conventional method, the pellet was resuspended in 1 mL of primary medium, seeded into a gelatin-coated 12-well dish, and incubated at 37°C and 5% CO_2 (day 0). After 24, 48, and 72 h, 1 mL primary medium was added to the 12-well dish. On day 4, the primary medium was removed leaving 1 mL, and 1 mL RE/MC proliferation medium was added. From day 5, half of the medium was replaced every day. For the combination method, the pellet was resuspended in 1 mL of primary medium containing 10 µM Y-27632 (Nacalai Tesque) and seeded onto Matrigel (Corning, Inc., Corning, New York, NY) coated (1:25) 12-well dish. The medium was changed in the same manner as in the conventional method by adding 10 µM Y-27632.

MEFs, CEFs, CDFs, and cUCs were passaged using 0.25% trypsin-EDTA (Sigma Aldrich) and stored at -80°C using a cell banker (Nippon Zenyaku Kogyo, Fukushima, Japan).

Reprogramming canine cells without feeder cells

When we reprogrammed canine cells with other feeder-free culture system, we altered DEF-CS to other feeder-free culture system, iMatrix-511 with StemFit or Vitronectin (VTN-N) Recombinant Human Protein, Truncated (Vitronectin; Thermo Fisher Scientific) with StemFlex (Thermo Fisher Scientific). Primary colonies were passaged onto iMatrix-511-coated dishes and maintained in StemFit.

ciPSC cryopreservation

ciPSCs were dissociated with TrypLE Select (Thermo Fisher Scientific) and stored at −80°C or −276°C using a stem cell banker (Nippon Zenyaku Kogyo, Fukushima, Japan).

RT-PCR and quantitative RT-PCR

Total RNA was extracted using the RNeasy Micro Kit (Qiagen, Hilden, Germany). Reverse transcription (RT) was performed using random primers and ReverTra Ace (Toyobo). Polymerase chain reaction (PCR) was performed using a Blend Taq Plus (Toyobo). PCR products were resolved on a 2% agarose gel stained with ethidium bromide and observed using an ultraviolet transilluminator (AE-9020; ATTO, Tokyo, Japan).

To quantify mRNA expression levels, PCR was performed using PowerTrack™ SYBR™ Green Master Mix (Thermo Fisher Scientific) and a QuantStudio 12K Flex real-time PCR machine (Theremo Fisher Scientific). *β-ACTIN* was used as a normalization control gene, and relative gene expression levels were calculated by ΔΔCt method[3]. All primers are listed in Table S2.

Immunocytochemistry

The cells were fixed in 4% paraformaldehyde, permeabilized with 0.1% Tween 20 in PBS (-) and blocked with 10% bovine serum albumin (Fujifilm Wako Pure Chemical Corporation). The cells were then incubated with primary antibodies at 4°C overnight. Negative control cells were incubated in PBS without primary antibodies. On the next day, the cells were washed, incubated with appropriate secondary antibodies at 20–25°C for 1 h, and mounted using ProLong Gold Antifade Reagent with 4′ ,6-diamidino-2-phenylindole (Thermo Fisher Scientific) to label DNA. Immunolabeled cells were observed using a confocal laser microscope (FV3000; Olympus, Tokyo, Japan). All antibodies are listed in Table S3.

Supplemental reference

1. Nakanishi, M., and Iijima, M. (2020). GENE EXPRESSION SYSTEM USING STEALTHY RNA, AND GENE INTRODUCTION/EXPRESSION VECTOR INCLUDING SAID RNA. United States Patent No. 10544431 B1.

2. Xu, Y., Zhang, T., Chen, Y., Shi, Q., Li, M., Qin, T., Hu, J., Lu, H., Liu, J., and Chen, C. (2020). Isolation and Characterization of multipotent canine urine-derived stem cells. Stem Cells Int. 2020, 8894449 10.1155/2020/8894449.

3. Livak, K.J., and Schmittgen, T.D. (2001). Analysis of relative gene expression data using real-time quantitatiive PCR and the 2−ΔΔCT method. Methods 25, 402–408. 10.1006/meth.2001.1262.
