# Supplementary material for: Generation of canine induced pluripotent stem cells under feeder-free conditions using Sendai virus vector encoding six canine reprogramming factors
Source: Stem Cell Reports. 2023 Dec 21;19(1):141–57. doi: 10.1016/j.stemcr.2023.11.010 (PMC10828825; doi:10.1016/j.stemcr.2023.11.010)
Supplement: Document S2. Article plus supplemental information [file mmc2.pdf]

Stem Cell Reports
Resource

OPEN ACCESS

Generation of canine induced pluripotent stem cells under feeder-free conditions using Sendai virus vector encoding six canine reprogramming factors

Masaya Tsukamoto,[1,2,3] Kazuto Kimura,[1,2] Takumi Yoshida,[1,2] Miyuu Tanaka,[4,5] Mitsuru Kuwamura,[4,5] Taro Ayabe,[6] Genki Ishihara,[6] Kei Watanabe,[6] Mika Okada,[7] Minoru Iijima,[7] Mahito Nakanishi,[7] Hidenori Akutsu,[3] Kikuya Sugiura,[1,2] and Shingo Hatoya[1,2,*]
[1]Department of Advanced Pathobiology, Graduate School of Veterinary Science, Osaka Metropolitan University, Izumisano, Osaka 598-8531, Japan
[2]Department of Advanced Pathobiology, Graduate School of Life and Environmental Sciences, Osaka Prefecture University, Izumisano, Osaka 598-8531, Japan
[3]Center for Regenerative Medicine, National Center for Child Health and Development, Setagaya, Tokyo 157-8535, Japan
[4]Department of Integrated Structural Biosciences, Graduate School of Veterinary Science, Osaka Metropolitan University, Izumisano, Osaka 598-8531, Japan
[5]Department of Integrated Structural Biosciences, Graduate School of Life and Environmental Sciences, Osaka Prefecture University, Izumisano, Osaka 598-8531, Japan
[6]Anicom Specialty Medical Institute, Shinjuku-ku, Tokyo 231-0033, Japan
[7]TOKIWA-Bio, Tsukuba, Ibaraki 305-0047, Japan
*Correspondence: hatoya@omu.ac.jp
https://doi.org/10.1016/j.stemcr.2023.11.010

SUMMARY

Although it is in its early stages, canine induced pluripotent stem cells (ciPSCs) hold great potential for innovative translational research in regenerative medicine, developmental biology, drug screening, and disease modeling. However, almost all ciPSCs were generated from fibroblasts, and available canine cell sources for reprogramming are still limited. Furthermore, no report is available to generate ciPSCs under feeder-free conditions because of their low reprogramming efficiency. Here, we reanalyzed canine pluripotency-associated genes and designed canine *LIN28A*, *NANOG*, *OCT3/4*, *SOX2*, *KLF4*, and *C-MYC* encoding Sendai virus vector, called 159cf. and 162cf. We demonstrated that not only canine fibroblasts but also canine urine-derived cells, which can be isolated using a noninvasive and straightforward method, were successfully reprogrammed with or without feeder cells. ciPSCs existed in undifferentiated states, differentiating into the three germ layers *in vitro* and *in vivo*. We successfully generated ciPSCs under feeder-free conditions, which can promote studies in veterinary and consequently human regenerative medicines.

INTRODUCTION

In humans, regenerative medicine using pluripotent stem cells (PSCs), including embryonic stem cells (ESCs) and induced pluripotent stem cells (iPSCs), is actively researched. Although rodents are frequently used for preclinical models, these models are not appropriate because of their short life spans and their living environment (Volk and Theoret, 2013). Canines, however, live in environments similar to those of humans, live longer than rodents, and develop spontaneously occurring diseases similar to humans (Hoffman et al., 2018), making them valuable and readily available preclinical models for human medicine (Kol et al., 2015).

Canine ESC generation is challenging because of an unusual breeding cycle and difficulty in *in vitro* maturation and *in vitro* fertilization of oocytes (Hatoya et al., 2006). In addition, as ESC generation has ethical concerns, canine iPSCs (ciPSCs) are a great cell source for regenerative medicine. Several researchers, including us, have reported ciPSC induction from fibroblasts introducing human *KLF4*, *OCT3/4*, *SOX2*, and *C-MYC* (Tsukamoto et al., 2018). However, low reprogramming efficiency limits the types of canine somatic cells available for iPSC induction. For clinical application, it is desirable to generate ciPSCs from various types of canine cells. In humans, urine-derived cells (UCs), easily and noninvasively isolated from urine samples, are an attractive cell source for iPSC induction (Zhou et al., 2012). Urine tests are common in the veterinary clinic. Therefore, generating ciPSCs from canine UCs (cUCs) would expand the potential for ciPSC application.

Although mouse embryonic fibroblasts (MEFs) were generally used as feeder cells to culture ciPSCs, they introduce variability in experimental conditions (Mallon et al., 2006) and increase the risk of pathogen transmission (DeSousa et al., 2006) and immune rejection (Martin et al., 2005). From these perspectives, ciPSCs should be generated and maintained under feeder-free conditions. We have reported that ciPSCs could be maintained in feeder-free conditions using StemFit medium and laminin 511 E8 fragment (Kimura et al., 2021b). However, all of the reported ciPSCs were established using MEFs. During the reprogramming of mouse and human cells under feeder-free conditions, the reprogramming efficiency decreased compared to that when using feeder cells (Sun et al., 2009). In canines,

This is an open access article under the CC BY license (http://creativecommons.org/licenses/by/4.0/).

the low reprogramming efficiency hinders the ciPSC induction under feeder-free conditions.

To reprogram various types of somatic cells under feeder-free conditions, we must improve reprogramming efficiency. The addition of *NANOG* and *LIN28A* to *KLF4*, *OCT3/4*, *SOX2*, and *C-MYC* has been reported to improve the reprogramming efficiency and enable the generation of human T cell–derived iPSCs (Honda et al., 2020). In addition, although somatic cells in various species can be reprogrammed using promiscuous pluripotency-associated factors (Nelson et al., 2009; Petkov et al., 2017), successful reprogramming may depend on the species providing the transcription factors (Lu et al., 2012; Ogorevc et al., 2016).

We hypothesized that the introduction of canine *KLF4*, *OCT3/4*, *SOX2*, *C-MYC*, *NANOG*, and *LIN28A* using Sendai virus vector (SeV) may improve canine cell reprogramming efficiency, leading to the generation of footprint-free ciPSCs from various cell types without feeder cells. We identified primary sequences of canine *KLF4*, *C-MYC*, and *NANOG* by deducing data from the CanFam3.1 database, which contains a canine reference genome obtained using Sanger sequencing (Lindblad-Toh et al., 2005). These sequences are significantly different from those found in humans and mice. In this study, we re-examined and determined putative sequences of canine reprogramming factors. We incorporated canine genes and generated a canine-specific SeV encoding six canine factors called 159cf. and 162cf. Using canine six factors SeV, we aimed to improve the reprogramming efficiency of canine cells and generate ciPSCs under feeder-free conditions.

RESULTS

Re-examination of primary sequence of canine reprogramming factors

We compared the gene sequences of canine *LIN28A*, *NANOG*, *OCT3/4*, *SOX2*, *KLF4*, and *C-MYC* with those in humans and mice using a genome database. The number of exons and introns in canine six genes were identical to those in humans and mice. Meanwhile, the start codons of canine *KLF4* and *C-MYC* were located 3′ to the human and mouse genes (Figure S1A) in the database. The 5′ rapid amplification of cDNA ends for canine *KLF4* and *C-MYC* revealed that the start codon of canine *KLF4* (ATG) is 27 bases upstream of that registered in CanFam3.1 similar to that in humans and other model animals; the start codon of canine *C-MYC* (CTG) is 39 bases upstream (Figure S1A). Our comparison using a genome database also revealed that the homology of *NANOG* between canines and humans was significantly lower than that of the other five reprogramming factors. Subsequently, we acquired partial sequences of these six canine factors by PCR and compared them with sequences in the database. We found a significant difference in the homology of *KLF4* (data not shown), suggesting that the predicted sequence of canine *KLF4* in CanFam3.1 could be incorrect.

Therefore, we resequenced the entire length of canine *KLF4* and *NANOG*. We found 89.50% homology at the DNA level and 86.56% homology at the amino acid level between our data (DNA Data Bank of Japan [DDBJ]: LC672616) and the predicted canine sequence (GenBank: XM_005626996) for *KLF4* (Figures S1B and S1C). KLF4 contains zinc-finger motifs at the C terminus and a transcriptional regulatory domain at the N terminus (Schuetz et al., 2011). The sequential differences in KLF4 between our data and predicted data were located from approximately 360 to 440 amino acids; this region may be part of the zinc-finger motifs. Our determined KLF4 sequence (DDBJ: LC672616) had only 86.88% homology with human KLF4 splicing variant 1 (GenBank: NM_001314052) (Figure S1D); however, it shared high homology (92.89%) with the human KLF4 splicing variant 2 (GenBank: NM_004235) (Figure S1E). This result indicated that the KLF4 determined in this study is comparable with human KLF4 splicing variant 2. To our knowledge, although splicing variants of mouse klf4 has different capacities for the maintenance of pluripotency in mouse ESCs (Yang et al., 2020), little is known about the functional differences in human KLF4 splicing variants in the regulation of pluripotency. We applied our sequence data as canine KLF4 for further study because human KLF4 splicing variant 2 (NM_004235) is traditionally transduced to reprogram somatic cells (Yamanaka et al., 2018). According to canine *NANOG*, although the sequence acquired in this study was identical to the predicted sequence from the database, the canine NANOG sequence determined in this study (DDBJ: LC672615) showed only 64.95% homology with human NANOG (GenBank: NM_024865) at the amino acid level (Figure S1F). The homologies of all of the stemness-associated genes between canines and humans are summarized in Figure S1G.

Reprogramming canine embryonic fibroblasts (CEFs) by expression of canine reprogramming factors with SeV

To achieve high reprogramming efficiency and produce high-quality iPSCs, the stoichiometry of multiple exogenous genes should be considered (Carey et al., 2011). The use of cytoplasmic RNA virus vectors necessitates the incorporation of all of the necessary genes onto a single vector to ensure their expression at a fixed stoichiometric ratio (Nishimura et al., 2011); owing to the importance of this requirement, we chose to use SeV carrying all six canine reprogramming genes (*OCT3/4*, *KLF4*, *SOX2*, *C-MYC*, *NANOG*, and *LIN28A*) in this order (Figure 1A). This order

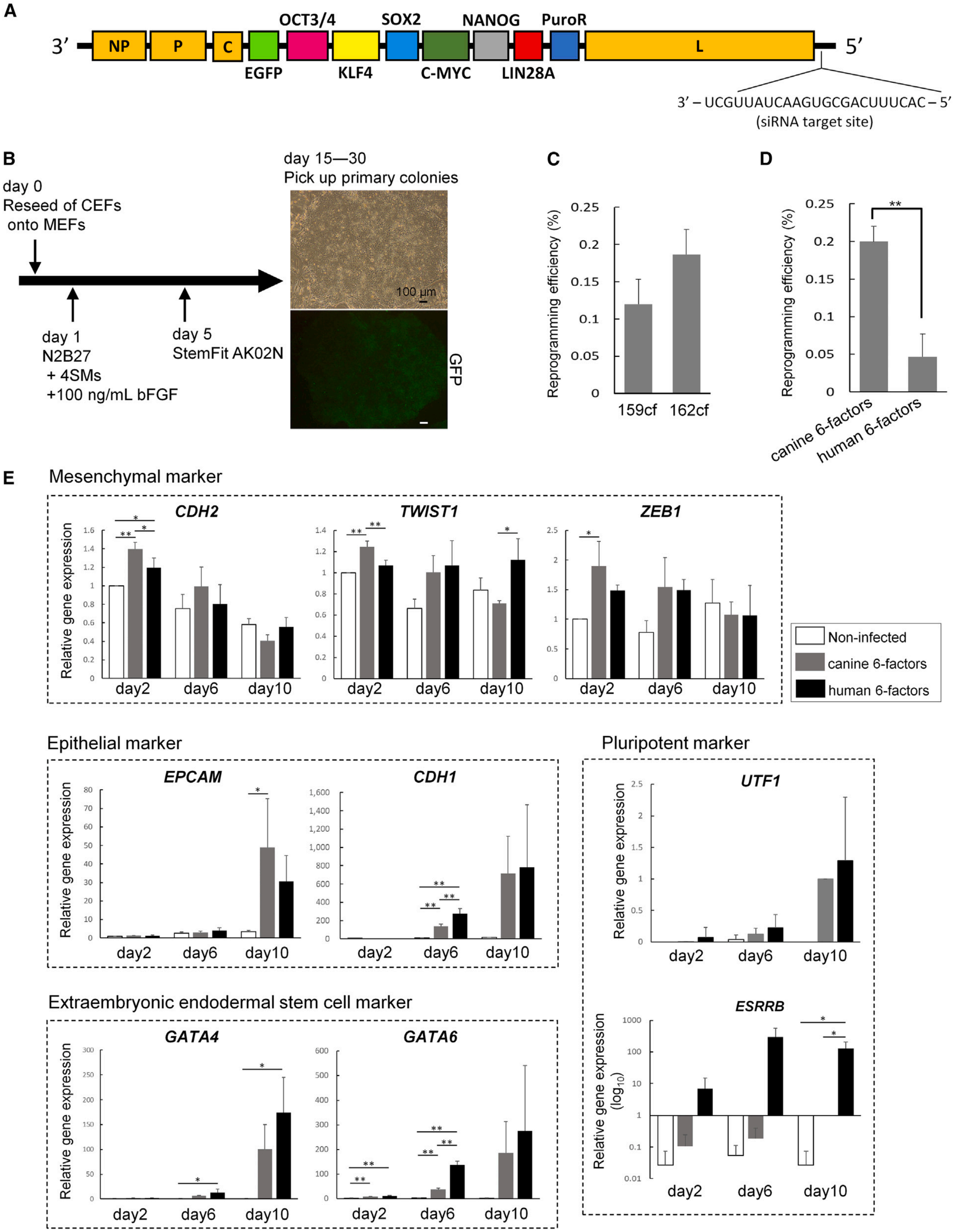

(legend on next page)

was optimal in human cell reprogramming and successfully used for reprogramming human T cells (Honda et al., 2020). Using canine-specific sequences, we also constructed two types of canine six factors SeV, 159cf. and 162cf., with different expression levels. The 159cf. expresses the exogenous genes at higher levels than 162cf. does, by controlling nucleocapsid protein expression (Nakanishi and Iijima, 2020). These vectors also contained EGFP and puromycin resistance genes (Figure 1A). Canine six factors SeV infected 33.7% of CEFs (n = 3) (Figure S1H). We confirmed that the canine six factors SeV expressed all six reprogramming factors and *EGFP* in infected CEFs, and 159cf. expressed exogeneous gene levels approximately 3-fold higher than those expressed by 162cf., according to qRT-PCR (Figure S1I).

To determine the reprogramming capacity of 159cf. and 162cf., we infected CEFs with canine six factors SeV. After reseeding onto MEFs, SeV-infected CEFs were cultured for 4 days in N2B27 medium containing a small-molecule cocktail that included MEK inhibitor PD0325901, GSK3β inhibitor CHIR99021, TGF-β inhibitor A-83-01, and Rock inhibitor Y27632 (4SMs) to promote cell reprogramming, as described in a previous report (Kimura et al., 2021a), with some modifications. Then, we cultured the cells in StemFit medium, as shown in Figure 1B. Approximately 10 days after SeV infection, primary colonies emerged using 159cf. and 162cf.; by day 30, the colonies were large enough to pick. The primary colonies were positive for EGFP (Figure 1B), indicating that they contained SeV. The average reprogramming efficiencies, based on the number of alkaline phosphatase–positive colonies, were 0.120% and 0.187% with 159cf. and 162cf., respectively. The reprogramming efficiency did not significantly differ between 159cf. and 162cf. (Figures 1C and S2A) ($p = 0.499$). These efficiencies were remarkably higher than those observed in our previous study (Tsukamoto et al., 2018), which was approximately 0.02% using the same CEFs as donor cells. When compared using the same methodology, the colony-forming efficiency using canine genes was higher than that using human genes (Figure 1D) ($p < 0.01$).

Sequential qPCR analysis revealed both canine and human gene–introduced cells underwent mesenchymal-to-epithelial transition during reprogramming (Figure 1E). Despite the similar expression level of the pluripotent marker *UTF1*, another pluripotency marker, *ESRRB*, was significantly upregulated in cells transduced with human genes (Figure 1E). *Esrrb* is linked to the differentiation of PSCs into extraembryonic endodermal stem (XEN) cells (Levy et al., 2022). Human gene–transduced cells also expressed higher levels of the XEN master regulator genes *GATA4* and *GATA6* (Fujikura et al., 2002; Levy et al., 2022) (Figure 1E), suggesting that human factors promoted direct reprograming of canine cells into XEN lineage rather than inducing pluripotency.

Primary colonies obtained using canine genes were subcultured onto iMatrix-511 and cultured in StemFit, as described previously (Kimura et al., 2021b), after which small interfering RNA (siRNA) was used to remove SeV. After siRNA treatment, some cells differentiated, whereas others became negative for EGFP (Figure S2B). After repeating the siRNA procedure 2–3 times, we generated EGFP$^-$subclones OPUiEF1-A-4, OPUiEF1-B-2, and OPUiEF1-C-3 using 162cf. and OPUiEF1-D-2 using 159cf. EGFP$^-$ subclones did not contain SeV (Figure 2A). Even after multiple passages (>50 times), they maintained their morphologies similar to primed PSCs, with clear borders and a high nucleus-to-cytoplasm ratio (Figures 2B and S2C). qPCR showed that these ciPSCs expressed similar levels of *OCT3/4* and *SOX2*, with varying *NANOG* levels among cell lines (Figure 2C). In addition, we quantitatively confirmed that none of the ciPSCs contained SeV (Figure S2D). Immunocytochemistry showed that ciPSCs expressed undifferentiated markers at the protein level (Figures 2D and S2E). After culturing the cells without basic fibroblast growth factor (bFGF), they demonstrated differentiation into the three germ layers *in vitro*, as detected by qPCR, RT-PCR, and immunocytochemistry (Figures 2E, 2F, S2F, and S2G). Two ciPSCs, OPUiEF1-A-4 and OPUiEF1-B-2, formed teratomas containing the three germ layers (Figure 2G). Furthermore, after at least 15 passages, ciPSCs

Figure 1. Reprogramming kinetics using canine six factors SeV

(A) Structure of SeV encoding 6 canine genes. In addition to reprogramming genes, SeV contained EGFP and puromycin-resistant genes (*PuroR*). An siRNA procedure was performed to remove SeV from reprogrammed cells.

(B) Scheme for reprogramming CEFs on MEFs. During cell reprogramming, small-molecule cocktails (4SMs) were added. The images at right show a primary colony morphology in bright field and its GFP expression. Black and white scale bar, 100 μm.

(C) Comparison of reprogramming efficiency using 159cf. and 162cf. The 2 groups did not differ significantly ($p = 0.499$).

(D) Comparison of reprogramming efficiency using canine six factors SeV (162cf.) and human six factors SeV. $^{**}p < 0.01$.

(E) Sequential qRT-PCR analysis during cell reprogramming using canine six factors SeV (gray bar) or human six factors SeV (black bar). SeV noninfected cells are shown as the control (white bar). *β-ACTIN* was used as a normalization control gene, and relative gene expression to noninfected cells on day 2 (except for pluripotent marker) or to canine six factors transduced cells on day 10 (pluripotent marker). $^{*}p < 0.05$; $^{**}p < 0.01$. Statistical significance was assessed between canine factor–transduced cells, human factor–transduced cells, and SeV noninfected cells at each time point (days 2, 6, and 10) using Tukey-Kramer test.

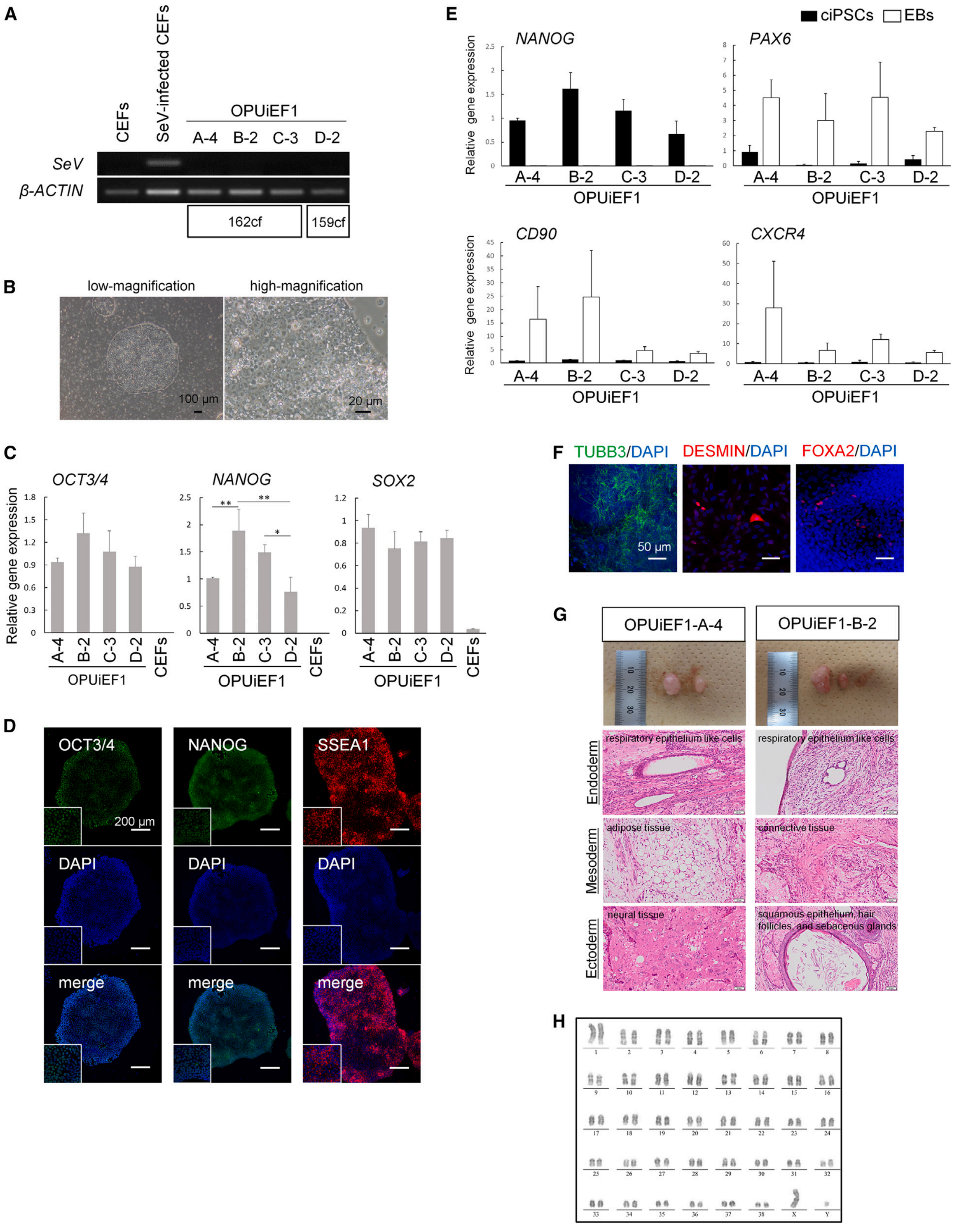

(legend on next page)

had normal 78 XX karyotypes or XY karyotypes, with 38 matched pairs of autosomes assessed by Q-banding (Figures 2H and S2H).

Generation of ciPSCs from canine adult cells by induction of canine six reprogramming factors

To examine the reprogramming capacity of adult cells, we infected canine dermal fibroblasts (CDFs) from two individuals with 159cf. or 162cf. We obtained no primary colonies under the conditions shown in Figure 1B. We recently reported that combining 4SMs with forskolin and ascorbic acid (6SMs) is beneficial for reprogramming canine peripheral blood mononuclear cells (Kimura et al., 2021a). After culturing infected CDFs with 6SMs for 4 days and then placing the cells in StemFit, as shown in Figure 3A, some primary colonies emerged on day 20 (Figure 3A). The reprogramming efficiency is shown in Figure S3A. After siRNA treatment, we generated SeV^{-}cell lines (from dog 1 using 159cf.: OPUiD05-FA-1 and OPUiD05-FB-3, and from dog 2 using 159cf.: OPUiD03-FA-3 and using 162cf.: OPUiD03-FB-3) (Figure 3B). We selected two ciPSC lines, OPUiD05-FA-1 and OPUiD03-FA-3, for detailed analysis. These cell lines did not contain SeV according to the results of qPCR (Figure S3B) and exhibited morphologies similar to primed iPSCs (Figures 3C and S3C). They expressed *OCT3/4* and *SOX2* transcripts at levels comparable to those in CEF-derived iPSCs, whereas *NANOG* expression varied among cell lines (Figure 3D). Immunostaining showed that both ciPSC lines expressed OCT3/4, NANOG, and SSEA1 (Figures 3E and S3D). Both ciPSCs demonstrated *in vitro* differentiation capacity, as assessed by qPCR, RT-PCR, and immunocytochemistry analysis (Figures 3F and S3E–S3G). Furthermore, both cell lines formed teratomas containing tissues from the 3 germ layers (Figure 3G) and had normal 78 XX karyotypes (Figures 3H and S3H).

Canine six factors SeV could reprogram CDFs into a pluripotent state, albeit the low efficiency state. In humans, UCs can be isolated using a noninvasive method and reprogrammed more efficiently than fibroblasts. We attempted to isolate cUCs using a method similar to that used in a previous study (Xu et al., 2020), here called the conventional method. However, we did not obtain iPSC colonies from cUCs (data not shown). The growth rate of somatic cells is an important index for successful reprogramming (Ruiz et al., 2011). A combination method of Matrigel and Y-27632 improved the isolation and expansion efficiency of cUCs from five dogs (Figures S4A and S4B), consistent with a previous study (Kim et al., 2020). These cUCs exhibited cobblestone morphologies similar to human UCs (Figure S4C), were positive for CD44, and were negative for CD34, CD45, and CD90 (Figure S4D). They expressed a high level of *SLC2A1* and low levels of *TWIST1* and *CD90* (Figure S4E), indicating derivation from the renal epithelium (Kim et al., 2020).

Reprogramming of cUCs derived by the combination method yielded primary colonies from four out of five dogs (dogs 1–5) (Figure 4A). We obtained no primary colonies from dog 5. The reprogramming efficiency varied depending on the individual (Figure S5A), as indicated previously (Xue et al., 2013). cUCs from certain individuals demonstrated exceptionally high reprogramming efficiency, with the highest efficiency reaching 2.43%. Resampling and reprogramming of cUCs from dog 5 resulted in some primary colonies using 162cf. (data not shown), suggesting that urine sample batch influences reprogramming kinetics, as reported in a previous study (Li et al., 2016). Our results indicated that the combination of MEFs and StemFit medium, which was developed for maintaining human PSCs without feeder cells, was effective for canine cell reprogramming, consistent with our previous study (Kimura et al., 2021a). Substituting StemFit with StemFlex, another

Figure 2. ciPSCs generated from CEFs using canine six factors SeV exhibited pluripotency

(A) RT-PCR showed that SeV was successfully removed from ciPSCs. CEFs and SeV-infected CEFs are shown as negative and positive controls, respectively. *β-ACTIN* was used as normalization control gene.

(B) Morphologies of OPUiEF1-A-4. Scale bar, 100 or 20 μm.

(C) qPCR for undifferentiated markers *OCT3/4*, *NANOG*, and *SOX2*. CEFs are shown as negative control, and *β-ACTIN* was used as an internal control. Relative gene expression to OPUiEF1-A-4. $^{*}p < 0.05$; $^{**}p < 0.01$.

(D) Immunocytochemistry of OPUiEF1-A-4 for pluripotent markers OCT3/4, NANOG, and SSEA1. Scale bar, 200 μm. High-magnification images are shown as insets.

(E) qPCR analysis of undifferentiated and differentiation markers 12 days after EB formation. Black bar and white bar represent ciPSCs and EBs, respectively. Undifferentiated marker *NANOG*, ectodermal marker *PAX6*, mesodermal marker *CD90*, and endodermal marker *CXCR4*. *β-ACTIN* was used as a normalization control gene.

(F) Immunocytochemistry for each differentiation marker after spontaneous differentiation of OPUiEF1-A-4. Ectodermal marker TUBB3, mesodermal marker DESMIN, and endodermal markers FOXA2. Scale bar, 50 μm.

(G) Teratoma formation of OPUiEF1-A-4 and B-2. The upper image shows the testis with a tumor (left) and normal testis (right). Teratomas contain the 3 germ layers: ectoderm; neural tissue or squamous epithelium, hair follicles, sebaceous glands, mesoderm; adipose tissue or connective tissue, and endoderm; respiratory epithelium-like cells. Scale bar, 50 μm.

(H) Karyotype analysis of OPUiEF1-A-4 at passage 14.

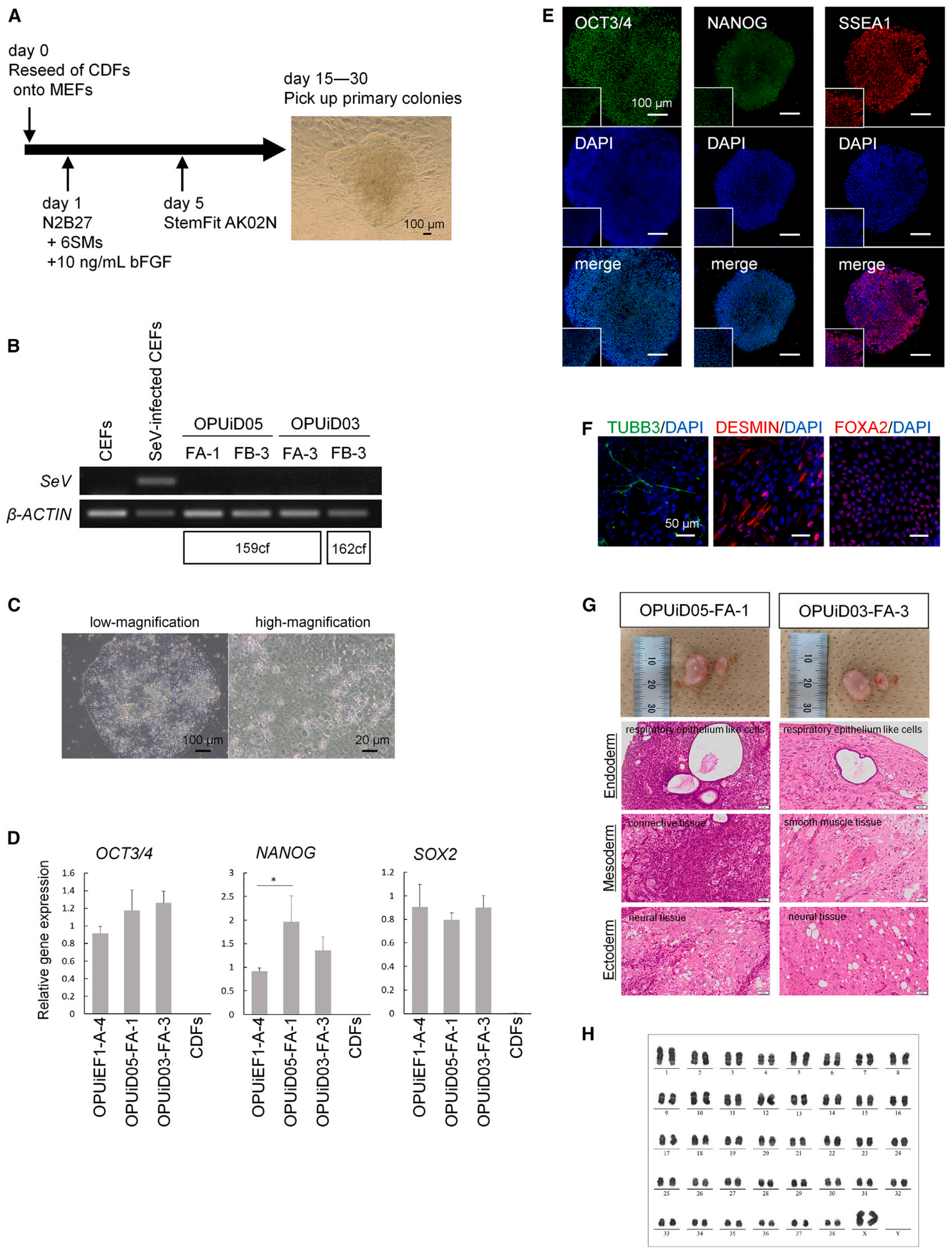

(legend on next page)

medium for feeder-free maintenance of human PSCs, resulted in primary colonies (Figure S5B), demonstrating the adaptability of our reprogramming method. We randomly picked up two primary colonies from each condition (individuals and vector types), and generated SeV$^-$ negative ciPSCs from cUCs isolated from all five dogs (Figure 4B).

We selected cUC-derived iPSCs using 162cf. from each individual, from dog 1: OPUiD06-UC-1, dog 2: OPUiD02-UD-1, dog 3: OPUiD01-UB-1, dog 4: OPUiD07-UC-3, and dog 5: OPUiD03-UB-1, for detailed characterization. None of the ciPSC lines contained SeV, as confirmed using qPCR (Figure S5C). The cells showed flat morphologies similar to fibroblast-derived ciPSCs (Figures 4C and S5D), and maintained these for >50 passages. All of the ciPSCs expressed *OCT3/4*, *NANOG*, and *SOX2* transcripts, albeit at varying levels (Figure 4D), and OCT3/4, NANOG, and SSEA1 proteins (Figures 4E and S5E). When cultured in the absence of bFGF, they differentiated into three germ layers *in vitro*, as detected using qPCR (Figure S5F), RT-PCR (Figure S5G), and immunocytochemistry (Figures 4F and S5H). Furthermore, OPUiD02-UD-1, OPUiD01-UB-1, and OPUiD07-UC-3 differentiated into three germ layers *in vivo* (Figure 4G), whereas other ciPSC lines differentiated into the ectoderm and mesoderm only (data not shown) (n = 2). All 5 ciPSC lines had normal 78 XX karyotypes (Figures 4H and S5I).

Generation of ciPSCs under feeder-free conditions using canine six Factors SeV

Canine six factors SeV reprogram canine somatic cells efficiently. Next, we attempted to reprogram 162cf.-infected CEFs, which were the same cells used for on-feeder reprogramming, under feeder-free conditions using combinations of iMatrix-511 with StemFit, vitronectin with StemFlex, or the Cellartis DEF-CS 500 Culture System. The DEF-CS system, which includes DEF-CS 500 medium and the coating reagent DEF-CS COAT1, has reportedly demonstrated high single-cell cloning efficiency for human PSCs (Chen et al., 2018; Gao et al., 2022). EGFP$^+$ primary colonies emerged at approximately 10 days after infection when cultured with the DEF-CS system without 4SMs (Figure 5A). Reprogramming efficiency was 0.01% (n = 2), which was lower than that with feeder cells (Figure 1C). Primary colonies were also obtained with iMatrix-511/StemFit or vitronectin/StemFlex when 4SMs were added for the first 4 days (Figure S6A). In all of the systems, fibroblasts reached confluence before colony emergence, which is consistent with a previous report (Tsukamoto et al., 2018). To inhibit the overgrowth of uninfected CEFs, we added puromycin.

Primary colonies were subcultured on iMatrix-511 and cultured in StemFit, establishing SeV$^-$ ciPSC lines OPUiEF1-E and OPUiEF1-F (Figures 5B and S6B). The cells exhibited flat morphologies (Figures 5C and S6C) and expressed undifferentiated markers according to qPCR (Figure 5D) and immunocytochemistry (Figures 5E and S6D). Other cell surface antigens were tested for two CEF-derived iPSCs, OPUiEF1-A-4 (reprogrammed using feeder cells) and OPUiEF1-E (reprogrammed under feeder-free conditions); these cells did not express SSEA4, TRA-1-60, and TRA-1-81 (Figure S6E). OPUiEF1-E and OPUiEF1-F exhibited the ability to differentiate into the three germ layers *in vitro* (Figures 5F, S6F, and S6G) and *in vivo* (Figure 5G), and had normal 78 XX karyotypes (Figures 5H and S6H).

Because cUCs derived using the combination method were reprogrammed with great efficiency, we then reprogrammed cUCs under feeder-free conditions using the DEF-CS system with or without 6SMs (Figure 6A). At approximately 15 days after reseeding, some primary colonies emerged (Figure 6A), with efficiency shown in Figure S6I. The colonies had less packed and more scattered

Figure 3. Generation of ciPSCs from CDFs and characteristics of CDF-derived iPSCs

(A) Scheme for reprogramming CDFs on MEFs. During the first 4 days, small-molecule cocktails (6SMs) were added to promote cell reprogramming. The image at right shows a primary colony morphology. Scale bar, 100 μm.

(B) RT-PCR of each ciPSC line for SeV. Amplifications of SeV were not detected in CDF-derived iPSCs from 2 individuals. CEFs and SeV-infected CEFs as negative and positive controls, respectively. *β-ACTIN* was used as a normalization control gene.

(C) Morphologies of OPUiD05-FA-1. Scale bar, 100 or 20 μm.

(D) qPCR for undifferentiated markers. CDFs are shown as negative control. *β-ACTIN* was used as an internal control, and relative gene expression to OPUiEF1-A-4, which was generated from CEFs. *$p < 0.05$.

(E) Immunocytochemistry of OPUiD05-FA-1 for pluripotent markers OCT3/4, NANOG, and SSEA1. Scale bar, 100 μm. High-magnification images are shown as insets.

(F) Immunocytochemistry for each differentiation marker after spontaneous differentiation of OPUiD05-FA-1. Ectodermal marker TUBB3, mesodermal marker DESMIN, and endodermal markers FOXA2. Scale bar, 50 μm.

(G) Teratoma formation of OPUiD05-FA-1 and OPUiD03-FA-3. The image at top shows the testis with a tumor (left) and normal testis (right). Teratomas contain the 3 germ layers: ectoderm; neural tissue, mesoderm; connective tissue or smooth muscle tissue, and endoderm; respiratory epithelium-like cells. Scale bar, 50 μm.

(H) Karyotype analysis of OPUiD05-FA-1 at passage 15.

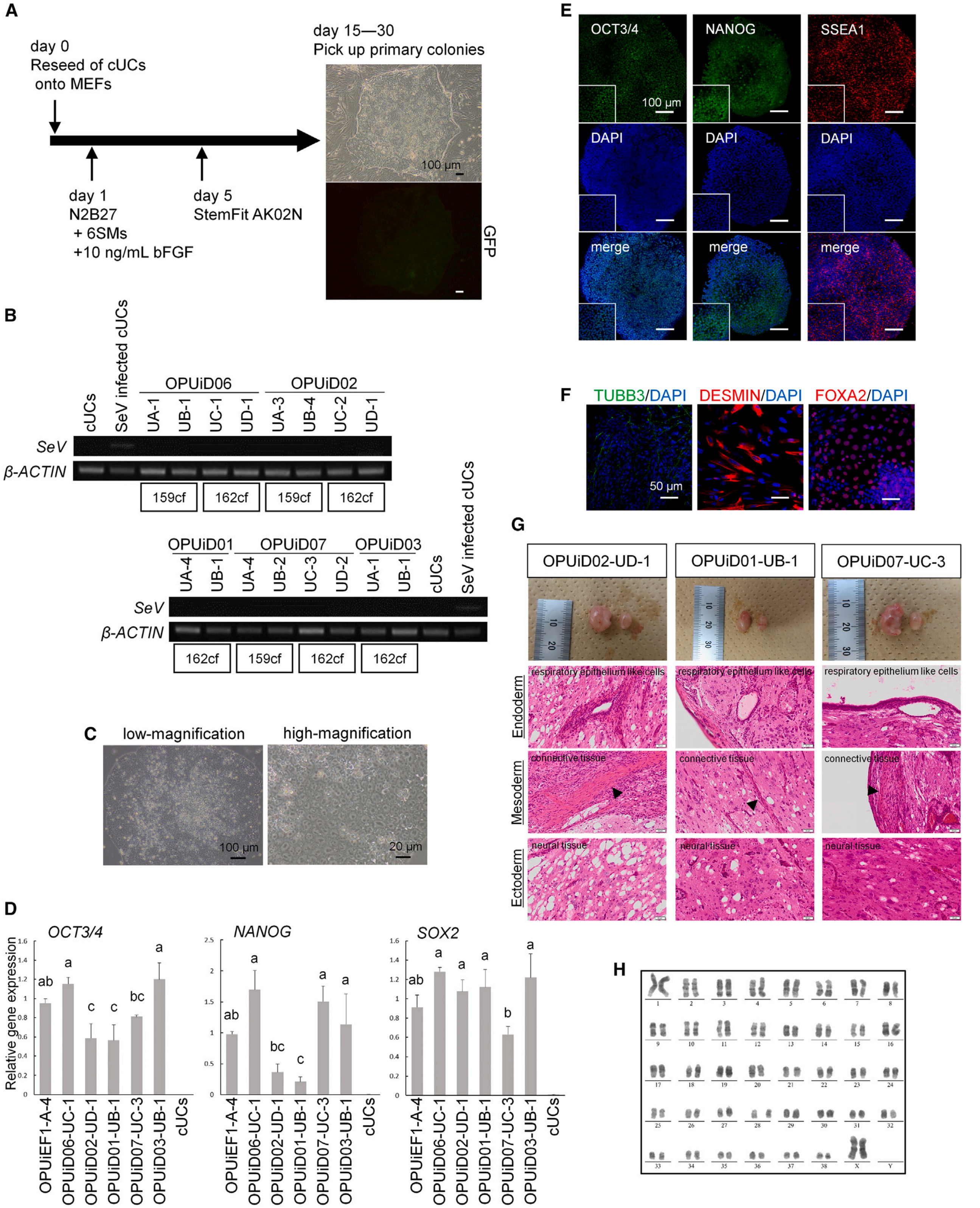

(legend on next page)

monolayer morphologies, which were atypical for human and canine PSCs, possibly due to the DEF-CS system (Figure 6A) (Chen et al., 2018). We obtained several primary colonies exhibiting typical PSC morphologies using iMatrix-511 and StemFit (Figure S6J). After siRNA treatment, SeV^{-} ciPSCs were generated from two dogs, dog 1: OPUiD06-UE-2 and dog 4: OPUiD07-UD-6 (Figures 6B and S6K), which resembled those derived from canine fibroblasts (Figures 6C and S6L) and expressed undifferentiation markers (Figures 6D, 6E, and S6M). OPUiD06-UE-2 spontaneously differentiated into the three germ layers *in vitro* without bFGF (Figures 6F, S6N, and S6O) and *in vivo* after testis capsule transplantation (Figure 6G). Although differentiation markers were upregulated when OPUiD07-UD-6 spontaneously differentiated under two-dimensional conditions (Figure S6O), it did not after embryoid body (EB) formation (Figures S6N and S6P). OPUiD07-UD-6 did not form teratomas (data not shown) (n = 2). Based on these results, OPUiD07-UD-6 is a differentiation-defective cell line. Both ciPSCs had normal 78 XX karyotypes (Figures 6H and S6Q). Information on the passage numbers obtained by characterizing all ciPSCs is shown in Table S1.

Collectively, we successfully reprogrammed canine cells under feeder-free conditions using canine six factors SeV, albeit the low reprogramming efficiency. Our newly established method allowed for efficient reprogramming of canine cells, leading to the generation of feeder-free ciPSCs from both CEFs and cUCs.

DISCUSSION

We resequenced and determined the full-length sequence of canine *KLF4* and *NANOG*, and generated SeV carrying canine *LIN28A*, *NANOG*, *OCT3/4*, *SOX2*, *KLF4*, and *C-MYC*. Using canine six factors SeV, we generated footprint-free and high-quality ciPSCs from CEFs, CDFs, and cUCs using feeder cells. Furthermore, we successfully generated ciPSCs under feeder-free conditions from CEFs and cUCs.

Using feeder cells, we generated ciPSCs stably from not only canine embryonic cells but also canine adult cells, as opposed to previous studies (Questa et al., 2020). One of the reasons for the successful reprogramming of canine adult cells may be the introduction of six canine factors. Although the effectiveness of species-specific reprogramming genes remains controversial (Lu et al., 2012; Ogorevc et al., 2016), we found that introducing six canine factors reprogrammed CEFs more efficiently than the six human factors. This could be because human factor transduction induced canine cells into XEN lineages rather than pluripotency. Comparison of the canine and human sequences revealed particularly low homology for *NANOG*. Because *NANOG* is beneficial in the late phase of reprogramming for the dedifferentiation of partially reprogrammed cells (Yu et al., 2007; Silva et al., 2009), canine *NANOG* may be more effective than human *NANOG* in canine cells.

Small molecules may also be important in the reprogramming process in canine cells because the addition of forskolin and ascorbic acid to 4SMs was necessary for CDF reprogramming. The components of 4SMs, GSK3β, and MEK inhibitors (Silva et al., 2008), TGF-β inhibition (Lin et al., 2009), and Rock inhibitor (Lai et al., 2010) were reported to promote reprogramming. However, reprogramming aged and terminally differentiated cells is more challenging than reprogramming embryonic cells in humans, mice, and canines (Wang et al., 2011; Trokovic et al., 2015; Questa et al., 2020). Ascorbic acid downregulates p16/Ink4a (Lee Chong et al., 2019), which is expressed at higher levels in aged cells and inhibits reprogramming (Li et al., 2009). Forskolin promotes the mesenchymal-to-epithelial transition and cell growth via the exchange factor directly

Figure 4. Generation of ciPSCs from cUCs and characteristics of cUC-derived iPSCs
(A) Scheme for reprogramming cUCs on MEFs. The images at right show a primary colony morphology in bright field and its GFP expression. Black and white scale bar, 100 μm.
(B) RT-PCR of each ciPSC line for SeV. There was no amplification of SeV in all of the cUC-derived iPSCs. cUCs and SeV-infected cUCs as negative and positive controls, respectively. *β-ACTIN* was used as a normalization control gene.
(C) Morphologies of OPUiD02-UD-1. Scale bar, 100 or 20 μm.
(D) qPCR for undifferentiated markers. cUCs are shown as negative control. *β-ACTIN* was used as an internal control, and relative gene expression to CEF-derived iPSCs, OPUiEF1-A-4. Groups labeled with different letters are significantly different from each other ($p < 0.05$).
(E) Immunocytochemistry of OPUiD02-UD-1 for pluripotent markers OCT3/4, NANOG, and SSEA1. Scale bar, 100 μm. High-magnification images are shown as insets.
(F) Immunocytochemistry of each differentiation marker after spontaneous differentiation of OPUiD02-UD-1. Ectodermal marker TUBB3, mesodermal marker DESMIN, and endodermal markers FOXA2. Scale bar, 50 μm.
(G) Teratoma formation of OPUiD02-UD-1, OPUiD01-UB-1, and OPUiD07-UC-3. The image at left shows the testis with a tumor (left) and normal testis (right). Teratomas contain the 3 germ layers: ectoderm; neural tissue, mesoderm; connective tissue (black arrowhead), and endoderm; respiratory epithelium-like cells. Scale bar, 50 μm.
(H) Karyotype analysis of OPUiD02-UD-1 at passage 15.

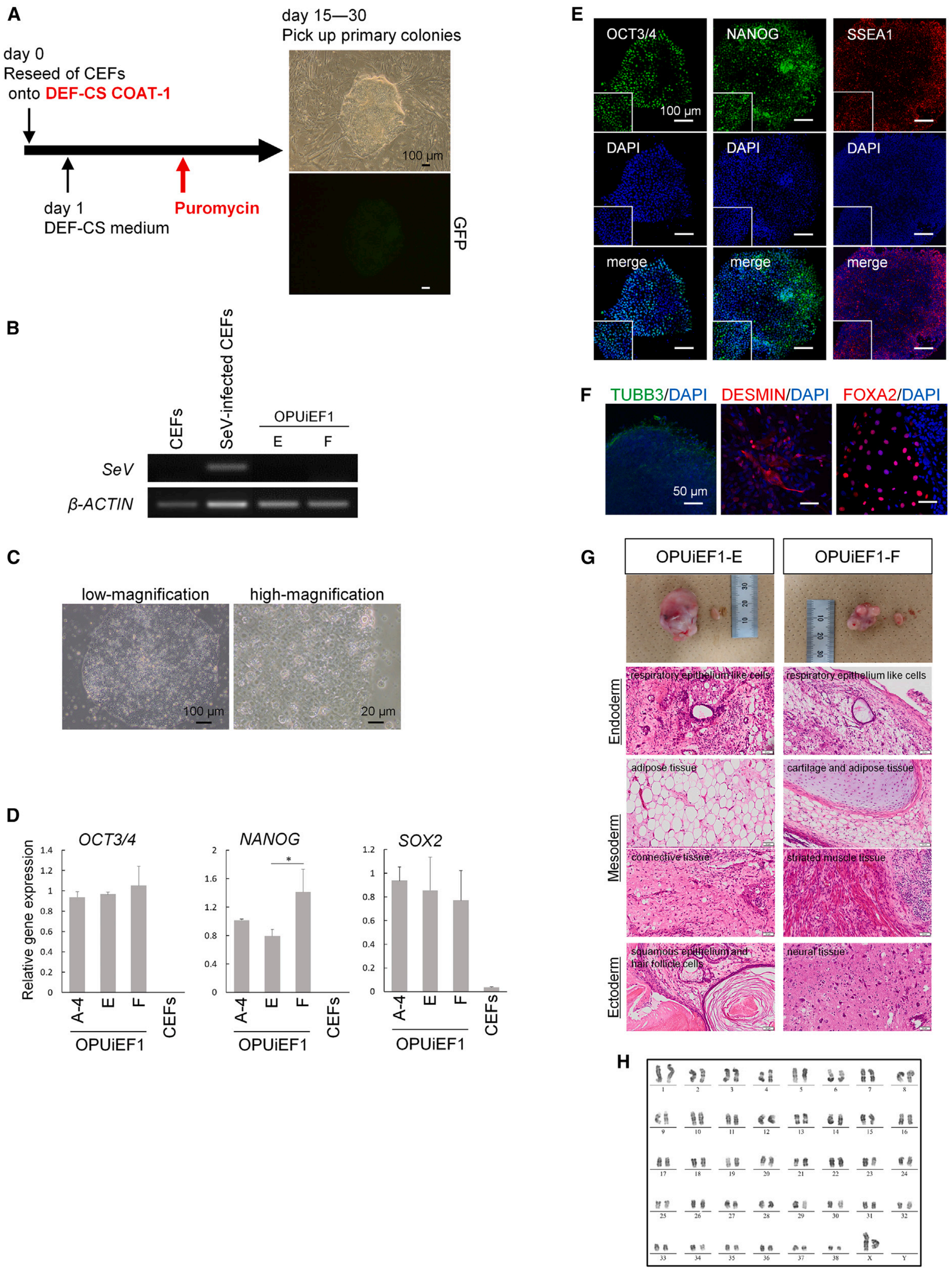

(legend on next page)

activated by cyclic AMP signaling, promoting the reprogramming process (Fritz et al., 2015). Thus, canine adult cells could be reprogrammed by adding 6SMs.

Under the same reprogramming conditions used for CDFs, cUCs were reprogrammed more easily than CDFs. Previous studies showed that human UCs are much easier to reprogram compared to fibroblasts because human UCs have epithelial properties and do not undergo a mesenchymal-to-epithelial transition (Xue et al., 2013), which is the main reprogramming barrier (Li et al., 2010). We showed that cUCs expressed a high level of *SLC2A1*, a renal epithelial marker (Kim et al., 2020). While we did not detail the cUCs phenotype, their potential epithelial properties could contribute to efficient reprogramming. Our data revealed that cUCs are an attractive cell source for generating ciPSCs due to their easy derivation without invasive methods and easy reprogramming.

The reported reprogramming efficiencies in mouse and human cells under feeder-free conditions are lower than those observed with feeder cells (Sun et al., 2009; Sugii et al., 2010). It is widely accepted that reprogramming canine somatic cells is more difficult compared to mouse and human somatic cells (Malik and Rao, 2013; Questa et al., 2020; Tsukamoto et al., 2020), which may explain the lack of effective methods for generating ciPSCs without feeder cells. In the present study, the use of canine six factors SeV enabled efficient reprogramming of canine cells and achieved ciPSC generation under feeder-free conditions. Feeder cells can cause variability in experimental conditions (Heng et al., 2004; Mallon et al., 2006), which is problematic for experimental reproducibility. Furthermore, in human regenerative medicine, a xeno-free system is required to reduce the risk of infection and immune rejection (Unger et al., 2008). Although there are no definite criteria in veterinary regenerative medicine, minimizing xenogeneic components is advisable considering their risks (Owens et al., 2016). Therefore, establishing a ciPSC induction method without feeder cells is essential. The ciPSCs generated in this study may be suitable for regenerative medicine. Our study demonstrated that reprogramming efficiency under feeder-free conditions was lower than that with feeder cells, which is consistent with previous findings in mouse and human cells. MEFs provide various factors that support pluripotency maintenance (Talbot et al., 2012). Therefore, conditions without MEFs may contribute to decreased reprogramming efficiency. Understanding in more detail the interactions between MEFs and canine cells that support pluripotency will be necessary to develop more effective methods for generating ciPSCs without the use of feeder cells.

Several studies have demonstrated that human iPSCs display inherent phenotypic and functional variations due to genetic or epigenetic differences, which can arise from donor cells or be acquired during cell reprogramming or extended culture (Liang and Zhang, 2013). Our study also found variations in pluripotent marker expression levels and differentiation potential among different ciPSC lines. Because all ciPSCs had normal karyotypes, aneuploidy can be ruled out as a cause for these variations. Moreover, donor genetic background may not cause these variations because distinct characteristics were observed in ciPSC lines from the same donor cells, such as OPUiD07-UC-3 and OPUiD07-UD-6. *De novo* variations obtained during cell reprogramming or extended culture may contribute to the phenotypic differences in ciPSCs, and further studies are needed to determine the phenotypic and functional variations in ciPSCs.

Stage-specific embryonic antigens (SSEAs) and tumor rejection antigens (TRAs) are expressed in stage- and species-specific manners. In human embryos, the morula expresses SSEA1 but not SSEA4 or TRA, whereas the inner cell mass in blastocysts expresses SSEA4 and TRA but not

Figure 5. Reprogramming CEFs under feeder-free conditions
(A) Scheme for reprogramming CEFs under feeder-free conditions. 162cf.-infected CEFs were seeded onto DEF-CS COAT-1-coated dishes and cultured in DEF-CS 500 medium. Before cells reached confluence, puromycin was added to the medium. The images at right illustrate primary colony morphology in bright field with GFP expression. Black and white scale bar, 100 μm.
(B) RT-PCR of 2 ciPSC lines for SeV. Amplifications of SeV were not observed in both ciPSC lines. CEFs and SeV-infected CEFs as negative and positive controls. *β-ACTIN* was used as a normalization control gene.
(C) Morphologies of ciPSCs, OPUiEF1-E. Scale bar, 100 or 20 μm.
(D) qPCR for undifferentiated markers. CEFs are shown as negative control. *β-ACTIN* was used as an internal control, and relative gene expression to ciPSCs derived from CEFs using feeder cells, OPUiEF1-A-4. *$p < 0.05$.
(E) Immunocytochemistry of OPUiEF1-E for pluripotent markers OCT3/4, NANOG, and SSEA1. Scale bar, 100 μm. High-magnification images are shown as insets.
(F) Immunocytochemistry for each differentiation marker of OPUiEF1-E after spontaneous differentiation. Ectodermal marker TUBB3, mesodermal marker DESMIN, and endodermal markers FOXA2. Scale bar, 50 μm.
(G) Teratoma formation of OPUiEF1-E and -F. Left, testis with a tumor; right, normal testis. Teratomas contain the 3 germ layers: ectoderm; neural tissue or squamous epithelium and hair follicle cells, mesoderm; adipose tissue, connective tissue, cartilage tissue, or striated muscle tissue, and endoderm; respiratory epithelium-like cells. Scale bar, 50 μm.
(H) Karyotype analysis of OPUiEF1-E at passage 17.

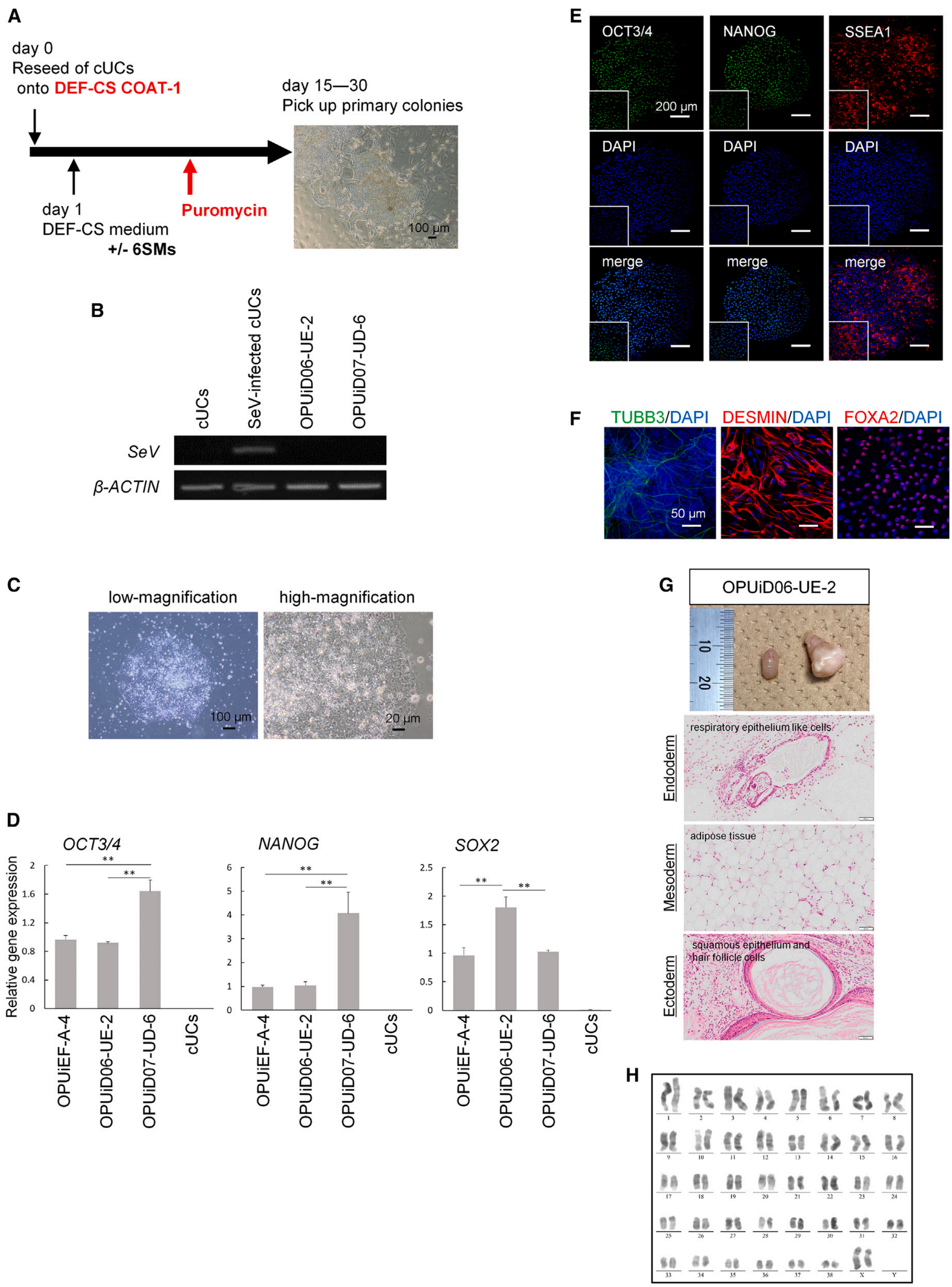

(legend on next page)

SSEA1. In mouse embryos, the morula expresses SSEA1 and SSEA4 but not TRA, whereas the inner cell mass expresses only SSEA1 (Henderson et al., 2002). The expression pattern of these antigens in canine embryos remains unknown because of the challenges in obtaining canine embryos (Hall et al., 2013). Although some researchers have generated canine ESCs, the expression pattern of these antigens varies among reports (Menon et al., 2019). In this study, we found that newly established ciPSCs expressed SSEA1 but not SSEA4, TRA-1-60, or TRA-1-81, consistent with our previous reports (Tsukamoto et al., 2018; Kimura et al., 2021a, 2021b). This suggests that the ciPSCs generated in this and previous studies corresponded to the same developmental stages during dog embryogenesis.

In conclusion, we generated footprint-free and high-quality ciPSCs using canine six factors SeV from not only CEFs and CDFs but also cUCs, which can be obtained using a simple and noninvasive method. Furthermore, we successfully generated ciPSCs from CEFs and cUCs under feeder-free conditions and reduced xenogeneic components during the induction of ciPSCs. Our method described in this study may facilitate veterinary regenerative medicine.

EXPERIMENTAL PROCEDURES

Resource availability

Corresponding author

Further information and requests for resources and reagents should be directed to and will be fulfilled by the corresponding author, Shingo Hatoya (hatoya@omu.ac.jp)

Materials availability

There are restrictions to the availability of ciPSC lines and canine six factors SeV due to the lack of an external centralized repository for its distribution and our need to maintain the stock. We are glad to share them with reasonable compensation by requestor for its processing and shipping.

Data and code availability

The accession numbers for canine *NANOG* and *KLF4* reported in this paper is DDBJ: LC672615 and LC672616, respectively.

Experimental

Animals and ethical statements

This study was approved by the Institutional Animal Experiment Committee of Osaka Prefecture University (permission nos: 20-61, 20-107, 20-108, 20-179, 21-61, 21-62, 21-63, and 21-64) and performed according to the Animal Experimentation Regulations of the Osaka Prefecture University.

SeV infection and reprogramming of canine somatic cells

CEFs, CDFs, and cUCs were incubated with canine six factors SeV 159cf., or 162cf. at a MOI of 1. Cells were reseeded onto an inactivated MEF-coated dish for reprogramming with feeder cells. The medium was changed to N2B27 medium containing small-molecule cocktails and later replaced with StemFit AK02N (StemFit; Ajinomoto, Tokyo, Japan) or StemFlex (Thermo Fisher Scientific, Waltham, MA).

For feeder-free reprogramming, SeV-infected cells were cultured using Cellartis DEF-CS 500 Culture System (DEF-CS; Takara, Shiga, Japan). Briefly, CEFs or cUCs were reseeded onto a DEF-CS COAT-1 (1:6)-coated dish and cultured in DEF-CS medium. Before the cells reached confluence, 5 μg/mL puromycin was added. During cUC reprogramming, 6SMs were added to the DEF-CS medium for the first 4 days.

ciPSCs were maintained with iMatrix-511 and StemFit and passaged as cell clumps.

siRNA procedure for removing SeV vector

To remove SeV, siRNA was applied 1 day after passage using RNAi MAX (Thermo Fisher Scientific) and repeated at every passage until $EGFP^{-}$ colonies emerged. The removal of SeV was checked using PCR analysis. The sequences of siRNA and primers for SeV are listed in Table S2.

Characterization of ciPSCs

The passage numbers when ciPSCs were characterized are summarized in Table S1.

Figure 6. Reprogramming of cUCs under feeder-free conditions

(A) Scheme for reprogramming of cUCs under feeder-free conditions. If cUCs were reprogrammed using 6SMs, 6SMs were added for the first 4 days and then withdrawn. Puromycin was added before cells reached confluence. The image at right illustrates primary colony morphology. Scale bar, 100 μm.

(B) RT-PCR of cUC-derived iPSCs generated under feeder-free conditions for SeV. cUCs and SeV-infected cUCs are used as negative and positive controls. *β-ACTIN* was used as a normalization control gene.

(C) Morphologies of ciPSCs, OPUiD06-UE-2. Scale bar, 100 or 20 μm.

(D) qPCR for undifferentiated markers. cUCs are shown as negative control. *β-ACTIN* was used as an internal control, and relative gene expression to CEF-derived OPUiEF1-A-4. **$p < 0.01$.

(E) Immunocytochemistry of OPUiD06-UE-2 for pluripotent markers OCT3/4, NANOG, and SSEA1. Scale bar, 200 μm. High-magnification images are shown as insets.

(F) Immunocytochemistry of differentiation markers after spontaneous differentiation of OPUiD06-UE-2. Ectodermal marker TUBB3, mesodermal marker DESMIN, and endodermal markers FOXA2. Scale bar, 50 μm.

(G) Teratoma formation of OPUiD06-UE-2. The upper image shows the testis with a tumor (right) and normal testis (left). Teratomas contain the 3 germ layers: ectoderm; squamous epithelium and hair follicle cells, mesoderm; adipose tissue, and endoderm; respiratory epithelium-like cells. Scale bar, 50 μm.

(H) Karyotype analysis of OPUiD06-UE-2 at passage 25.

Alkaline phosphatase staining

ciPSCs were fixed in 4% paraformaldehyde and stained with Alkaline Phosphatase Staining Kit II (Stemgent, San Diego, CA) according to the manufacturer's instructions.

In vitro *differentiation assay*

The *in vitro* differentiation ability of ciPSCs was evaluated via EB formation or two-dimensional growth. To form EBs, ciPSCs were dissociated into cell clumps or single cells and cultured in Ultra-Low Attachment Plates (Corning Inc., Corning, NY) containing 20% fetal bovine serum (FBS) medium. After seven days, EBs were transferred to gelatin-coated slides and cultured in 20% FBS medium. At 14–20 days after seeding, the attached cells were fixed and immunolabeled. Antibodies are listed in Table S3. Alternatively, dissociated ciPSCs were seeded in Costar Ultra Low Cluster 96 Well Round Bottom Plate (Corning Inc.) at a density of 1.0×10^4 cells per well in 20% FBS medium, and maintained for 12 days. Subsequently, RNA was extracted, and qPCR was performed.

For two-dimensional differentiation, ciPSCs were cultured in StemFit without solution C for 2–3 days and then in 20% FBS medium for 7 days. RNA was extracted, and RT-PCR was performed.

Teratoma formation assay for assessing in vivo *differentiation ability*

Approximately 1×10^6 ciPSCs were injected into the testis capsule of NOD/SCID mice (n = 2 for each cell line). The mice were euthanized by cervical dislocation after 3 months, and the tumors were fixed, paraffin-embedded, sectioned, and stained with H&E.

Karyotyping analysis

ciPSCs were incubated with 0.05 μg/mL colcemid (Thermo Fisher Scientific), trypsinized, and incubated with 0.075 M KCl. The cells were then fixed in acetic acid:methanol (1:3), stained with quinacrine mustard and Hoechst 33258, and observed using confocal laser microscopy (LSM980; Carl Zeiss, Oberkochen, Germany).

Statistical analysis

Each experiment was performed three times independently as biological replicates. All of the data are expressed as the mean ± SD. Statistical significance was assessed by Student's t test or Tukey-Kramer multiple comparison using SPSS software (version 25; IBM SPSS Statistics, Armonk, NY).

Other experimental procedures are described in the supplemental experimental procedures.

SUPPLEMENTAL INFORMATION

Supplemental information can be found online at https://doi.org/10.1016/j.stemcr.2023.11.010.

ACKNOWLEDGMENTS

This work was supported by JSPS KAKENHI grant nos. JP18K19273, JP18H02349, JP19J22851, and JP22H02525. This work was also supported by JST Adaptable and Seamless Technology Transfer Program through Target-Driven R&D (A-STEP) grant no. JPMJTM20QH. This study was also funded by Anicom Specialty Medical Institute. This research was supported in part by the 2022 Osaka Metropolitan University (OMU) Strategic Research Promotion Project (Priority Research). We thank Manami Ohtaka for providing the reprogramming vector. We thank Editage (www.editage.com) for English-language editing.

AUTHOR CONTRIBUTIONS

Conception and design, M. Tsukamoto, G.I., M.N., and S.H.; data collection, M. Tsukamoto, K.K., and K.W.; data assembly, M. Tsukamoto, M. Tanaka, K.W., and M.N.; data analysis and interpretation, M. Tsukamoto, T.Y., M. Tanaka, M.K., T.A., K.W., and M.N.; manuscript writing, M. Tsukamoto and K.W.; final approval of the manuscript, M.K., M.N., H.A., K.S., and S.H.; administrative support, G.I., M.N., K.S., and S.H.; provision of study material or patients, G.I., M.O., and M.I.; financial support, G.I., M.N., K.S., and S.H.

DECLARATION OF INTERESTS

This study was also funded by Anicom Specialty Medical Institute. T.A. and K.W. are employees of Anicom Specialty Medical Institute. M.I. and M.N. are the inventors of the patent application (JP 6770224 B2, JP 7174958 B2, US 10544431 B1, EP 3246406 B1, TW I 696700 B, CN 107109400 B, SG 11201705820U, KR 6102459458 B1).

Received: November 14, 2022
Revised: November 27, 2023
Accepted: November 27, 2023
Published: December 21, 2023

REFERENCES

Carey, B.W., Markoulaki, S., Hanna, J.H., Faddah, D.A., Buganim, Y., Kim, J., Ganz, K., Steine, E.J., Cassady, J.P., Creyghton, M.P., et al. (2011). Reprogramming factor stoichiometry influences the epigenetic state and biological properties of induced pluripotent stem cells. Cell Stem Cell *9*, 588–598.

Chen, Y.H., and Pruett-Miller, S.M. (2018). Improving single-cell cloning workflow for gene editing in human pluripotent stem cells. Stem Cell Res. *31*, 186–192.

De Sousa, P.A., Galea, G., and Turner, M. (2006). The road to providing human embryo stem cells for therapeutic use: The UK experience. Reproduction *132*, 681–689.

Fritz, A.L., Adil, M.M., Mao, S.R., and Schaffer, D.V. (2015). cAMP and EPAC signaling functionally replace OCT4 during induced pluripotent stem cell reprogramming. Mol. Ther. *23*, 952–963.

Fujikura, J., Yamato, E., Yonemura, S., Hosoda, K., Masui, S., Nakao, K., Miyazaki Ji, J.i., and Niwa, H. (2002). Differentiation of embryonic stem cells is induced by GATA factors. Genes Dev. *16*, 784–789.

Gao, X., Sprando, R.L., and Yourick, J.J. (2022). Rapid and highly efficient isolation and purification of human induced pluripotent stem cells. Methods Mol. Biol. *2429*, 3–14.

Hall, V., Hinrichs, K., Lazzari, G., Betts, D.H., and Hyttel, P. (2013). Early embryonic development, assisted reproductive technologies, and pluripotent stem cell biology in domestic mammals. Vet. J. *197*, 128–142.

Hatoya, S., Torii, R., Kondo, Y., Okuno, T., Kobayashi, K., Wijewardana, V., Kawate, N., Tamada, H., Sawada, T., Kumagai, D., et al.

(2006). Isolation and characterization of embryonic stem-like cells from canine blastocysts. Mol. Reprod. Dev. *73*, 298–305.

Henderson, J.K., Draper, J.S., Baillie, H.S., Fishel, S., Thomson, J.A., Moore, H., and Andrews, P.W. (2002). Preimplantation human embryos and embryonic stem cells show comparable expression of stage-specific embryonic antigens. Stem Cell. *20*, 329–337.

Heng, B.C., Liu, H., and Cao, T. (2004). Feeder cell density–A key parameter in human embryonic stem cell culture. In Vitro Cell. Dev. Biol. Anim. *40*, 255–257.

Hoffman, J.M., Creevy, K.E., Franks, A., O'Neill, D.G., and Promislow, D.E.L. (2018). The companion dog as a model for human aging and mortality. Aging Cell *17*, e12737.

Honda, T., Ando, M., Ando, J., Ishii, M., Sakiyama, Y., Ohara, K., Toyota, T., Ohtaka, M., Masuda, A., Terao, Y., et al. (2020). Sustainable tumor-suppressive effect of iPSC-derived rejuvenated T cells targeting cervical cancers. Mol. Ther. *28*, 2394–2405.

Kim, K., Gil, M., Dayem, A.A., Choi, S., Kang, G.H., Yang, G.M., Cho, S., Jeong, Y., Kim, S.J., Seok, J., et al. (2020). Improved isolation and culture of urine-derived stem cells (USCs) and enhanced production of immune cells from the USC-derived induced pluripotent stem cells. J. Clin. Med. *9*, 827.

Kimura, K., Tsukamoto, M., Tanaka, M., Kuwamura, M., Ohtaka, M., Nishimura, K., Nakanishi, M., Sugiura, K., and Hatoya, S. (2021a). Efficient reprogramming of canine peripheral blood mononuclear cells into induced pluripotent stem cells. Stem Cell. Dev. *30*, 79–90.

Kimura, K., Tsukamoto, M., Yoshida, T., Tanaka, M., Kuwamura, M., Ohtaka, M., Nishimura, K., Nakanishi, M., Sugiura, K., and Hatoya, S. (2021b). Canine induced pluripotent stem cell maintenance under feeder-free and chemically-defined conditions. Mol. Reprod. Dev. *88*, 395–404.

Kol, A., Arzi, B., Athanasiou, K.A., Farmer, D.L., Nolta, J.A., Rebhun, R.B., Chen, X., Griffiths, L.G., Verstraete, F.J.M., Murphy, C.J., and Borjesson, D.L. (2015). Companion animals: Translational scientist's new best friends. Sci. Transl. Med. *7*, 308ps21.

Lai, W.H., Ho, J.C.Y., Lee, Y.K., Ng, K.M., Au, K.W., Chan, Y.C., Lau, C.P., Tse, H.F., and Siu, C.W. (2010). ROCK inhibition facilitates the generation of human-induced pluripotent stem cells in a defined, feeder-and serum-free system. Cell. Reprogr. *12*, 641–653.

Lee Chong, T., Ahearn, E.L., and Cimmino, L. (2019). Reprogramming the epigenome with vitamin C. Front. Cell Dev. Biol. *7*, 128.

Levy, S.H., Cohen, S.F., Arnon, L., Lahav, S., Awawdy, M., Alajem, A., Bavli, D., Sun, X., Buganim, Y., and Ram, O. Esrrb is a cell-cycle-dependent associated factor balancing pluripotency and XEN differentiation. Stem Cell Rep. 17, 1334–1350 .

Li, D., Wang, L., Hou, J., Shen, Q., Chen, Q., Wang, X., Du, J., Cai, X., Shan, Y., Zhang, T., et al. (2016). Optimized approaches for generation of integration-free iPSCs from human urine-derived cells with small molecules and autologous feeder. Stem Cell Rep. *6*, 717–728.

Li, H., Collado, M., Villasante, A., Strati, K., Ortega, S., Cañamero, M., Blasco, M.A., and Serrano, M. (2009). The Ink4/Arf locus is a barrier for iPS cell reprogramming. Nature *460*, 1136–1139.

Li, R., Liang, J., Ni, S., Zhou, T., Qing, X., Li, H., He, W., Chen, J., Li, F., Zhuang, Q., et al. (2010). A mesenchymal-to-epithelial transition initiates and is required for the nuclear reprogramming of mouse fibroblasts. Cell Stem Cell *7*, 51–63.

Liang, G., and Zhang, Y. (2013). Genetic and epigenetic variations in iPSCs: potential causes and implications for application. Cell Stem Cell *13*, 149–159.

Lin, T., Ambasudhan, R., Yuan, X., Li, W., Hilcove, S., Abujarour, R., Lin, X., Hahm, H.S., Hao, E., Hayek, A., and Ding, S. (2009). A chemical platform for improved induction of human iPSCs. Nat. Methods *6*, 805–808.

Lindblad-Toh, K., Wade, C.M., Mikkelsen, T.S., Karlsson, E.K., Jaffe, D.B., Kamal, M., Clamp, M., Chang, J.L., Kulbokas, E.J., III, Zody, M.C., et al. (2005). Genome sequence, comparative analysis and haplotype structure of the domestic dog. Nature *438*, 803–819.

Lu, Y., West, F.D., Jordan, B.J., Mumaw, J.L., Jordan, E.T., Gallegos-Cardenas, A., Beckstead, R.B., and Stice, S.L. (2012). Avian-induced pluripotent stem cells derived using human reprogramming factors. Stem Cell. Dev. *21*, 394–403.

Malik, N., and Rao, M.S. (2013). A review of the methods for human iPSC derivation. Methods Mol. Biol. *997*, 23–33.

Mallon, B.S., Park, K.Y., Chen, K.G., Hamilton, R.S., and McKay, R.D.G. (2006). Toward xeno-free culture of human embryonic stem cells. Int. J. Biochem. Cell Biol. *38*, 1063–1075.

Martin, M.J., Muotri, A., Gage, F., and Varki, A. (2005). Human embryonic stem cells express an immunogenic nonhuman sialic acid. Nat. Med. *11*, 228–232.

Menon, D.V., Patel, D., Joshi, C.G., and Kumar, A. (2019). The road less travelled: The efficacy of canine pluripotent stem cells. Exp. Cell Res. *377*, 94–102.

Nakanishi, M., and Iijima, M. (2020). GENE EXPRESSION SYSTEM USING STEALTHY RNA, and GENE INTRODUCTION/ EXPRESSION VECTOR INCLUDING SAID RNA. United States Patent No. 10544431 B1.

Nelson, T.J., Martinez-Fernandez, A., Yamada, S., Mael, A.A., Terzic, A., and Ikeda, Y. (2009). Induced pluripotent reprogramming from promiscuous human stemness related factors. Clin. Transl. Sci. *2*, 118–126.

Nishimura, K., Sano, M., Ohtaka, M., Furuta, B., Umemura, Y., Nakajima, Y., Ikehara, Y., Kobayashi, T., Segawa, H., Takayasu, S., et al. (2011). Development of defective and persistent Sendai virus vector: a unique gene delivery/expression system ideal for cell reprogramming. J. Biol. Chem. *286*, 4760–4771.

Ogorevc, J., Orehek, S., and Dovč, P. (2016). Cellular reprogramming in farm animals: An overview of iPSC generation in the mammalian farm animal species. J. Anim. Sci. Biotechnol. *7*, 10.

Owens, S.D., Kol, A., Walker, N.J., and Borjesson, D.L. (2016). Allogeneic mesenchymal stem cell treatment induces specific alloantibodies in horses. Stem Cell. Int. *2016*, 5830103.

Petkov, S.G., Glage, S., and Niemann, H. (2017). Mouse iPSC generated with porcine reprogramming factors as a model for studying the effects of non-silenced heterologous transgenes on pluripotency. J. Stem Cells Regen. Med. *13*, 20–28.

Questa, M., Moshref, M., Jimenez, R.J., Lopez-Cervantes, V., Crawford, C.K., Settles, M.L., Ross, P.J., and Kol, A. (2020). Chromatin accessibility in canine stromal cells and its implications for canine somatic cell reprogramming. Stem Cells Transl. Med. *10*, 441–454.

Ruiz, S., Panopoulos, A.D., Herrerías, A., Bissig, K.D., Lutz, M., Berggren, W.T., Verma, I.M., and Izpisua Belmonte, J.C. (2011). A high proliferation rate is required for cell reprogramming and maintenance of human embryonic stem cell identity. Curr. Biol. *21*, 45–52.

Schuetz, A., Nana, D., Rose, C., Zocher, G., Milanovic, M., Koenigsmann, J., Blasig, R., Heinemann, U., and Carstanjen, D. (2011). The structure of the Klf4 DNA-binding domain links to self-renewal and macrophage differentiation. Cell. Mol. Life Sci. *68*, 3121–3131.

Silva, J., Barrandon, O., Nichols, J., Kawaguchi, J., Theunissen, T.W., and Smith, A. (2008). Promotion of reprogramming to ground state pluripotency by signal inhibition. PLoS Biol. *6*, e253.

Silva, J., Nichols, J., Theunissen, T.W., Guo, G., van Oosten, A.L., Barrandon, O., Wray, J., Yamanaka, S., Chambers, I., and Smith, A. (2009). Nanog is the gateway to the pluripotent ground state. Cell *138*, 722–737.

Sugii, S., Kida, Y., Kawamura, T., Suzuki, J., Vassena, R., Yin, Y.Q., Lutz, M.K., Berggren, W.T., Izpisúa Belmonte, J.C., and Evans, R.M. (2010). Human and mouse adipose-derived cells support feeder-independent induction of pluripotent stem cells. Proc. Natl. Acad. Sci. USA *107*, 3558–3563.

Sun, N., Panetta, N.J., Gupta, D.M., Wilson, K.D., Lee, A., Jia, F., Hu, S., Cherry, A.M., Robbins, R.C., Longaker, M.T., and Wu, J.C. (2009). Feeder-free derivation of induced pluripotent stem cells from adult human adipose stem cells. Proc. Natl. Acad. Sci. USA *106*, 15720–15725.

Talbot, N.C., Sparks, W.O., Powell, A.M., Kahl, S., and Caperna, T.J. (2012). Quantitative and semiquantitative immunoassay of growth factors and cytokines in the conditioned medium of STO and CF-1 mouse feeder cells. In Vitro Cell. Dev. Biol. Anim. *48*, 1–11.

Trokovic, R., Weltner, J., Noisa, P., Raivio, T., and Otonkoski, T. (2015). Combined negative effect of donor age and time in culture on the reprogramming efficiency into induced pluripotent stem cells. Stem Cell Res. *15*, 254–262.

Tsukamoto, M., Kimura, K., Tanaka, M., Kuwamura, M., Ohtaka, M., Nakanishi, M., Sugiura, K., and Hatoya, S. (2020). Generation of footprint-free canine induced pluripotent stem cells from peripheral blood mononuclear cells using Sendai virus vector. Mol. Reprod. Dev. *87*, 663–665.

Tsukamoto, M., Nishimura, T., Yodoe, K., Kanegi, R., Tsujimoto, Y., Alam, M.E., Kuramochi, M., Kuwamura, M., Ohtaka, M., Nishimura, K., et al. (2018). Generation of footprint-free canine induced pluripotent stem cells using auto-erasable Sendai virus vector. Stem Cell. Dev. *27*, 1577–1586.

Unger, C., Skottman, H., Blomberg, P., Dilber, M.S., and Hovatta, O. (2008). Good manufacturing practice and clinical-grade human embryonic stem cell lines. Hum. Mol. Genet. *17*, R48–R53.

Volk, S.W., and Theoret, C. (2013). Translating stem cell therapies: The role of companion animals in regenerative medicine. Wound Repair Regen. *21*, 382–394.

Wang, B., Miyagoe-Suzuki, Y., Yada, E., Ito, N., Nishiyama, T., Nakamura, M., Ono, Y., Motohashi, N., Segawa, M., Masuda, S., and Takeda, S. (2011). Reprogramming efficiency and quality of induced pluripotent stem cells (iPSCs) generated from muscle-derived fibroblasts of mdx mice at different ages. PLoS Curr. *3*, RRN1274.

Xu, Y., Zhang, T., Chen, Y., Shi, Q., Li, M., Qin, T., Hu, J., Lu, H., Liu, J., and Chen, C. (2020). Isolation and Characterization of multipotent canine urine-derived stem cells. Stem Cell. Int. *2020*, 8894449.

Xue, Y., Cai, X., Wang, L., Liao, B., Zhang, H., Shan, Y., Chen, Q., Zhou, T., Li, X., Hou, J., et al. (2013). Generating a non-integrating human induced pluripotent stem cell bank from urine-derived cells. PLoS One *8*, e70573.

Yamanaka, S., Takahashi, K., and Nakagawa, M. (2018). Nuclear Reprogramming Factor and Induced Pluripotent Stem Cells. United States Patent US20180371036A1.

Yang, Y., Xiong, J., Wang, J., Ruan, Y., Zhang, J., Tian, Y., Wang, J., Liu, L., Cheng, Y., Wang, X., et al. (2020). Novel alternative splicing variants of Klf4 display different capacities for self-renewal and pluripotency in mouse embryonic stem cells. Biochem. Biophys. Res. Commun. *532*, 377–384.

Yu, J., Vodyanik, M.A., Smuga-Otto, K., Antosiewicz-Bourget, J., Frane, J.L., Tian, S., Nie, J., Jonsdottir, G.A., Ruotti, V., Stewart, R., et al. (2007). Induced pluripotent stem cell lines derived from human somatic cells. Science *318*, 1917–1920.

Zhou, T., Benda, C., Dunzinger, S., Huang, Y., Ho, J.C., Yang, J., Wang, Y., Zhang, Y., Zhuang, Q., Li, Y., et al. (2012). Generation of human induced pluripotent stem cells from urine samples. Nat. Protoc. *7*, 2080–2089.

Stem Cell Reports, Volume *19*

Supplemental Information

Generation of canine induced pluripotent stem cells under feeder-free conditions using Sendai virus vector encoding six canine reprogramming factors

Masaya Tsukamoto, Kazuto Kimura, Takumi Yoshida, Miyuu Tanaka, Mitsuru Kuwamura, Taro Ayabe, Genki Ishihara, Kei Watanabe, Mika Okada, Minoru Iijima, Mahito Nakanishi, Hidenori Akutsu, Kikuya Sugiura, and Shingo Hatoya

Figure S1, Canine sequence and canine 6-factors SeV

A

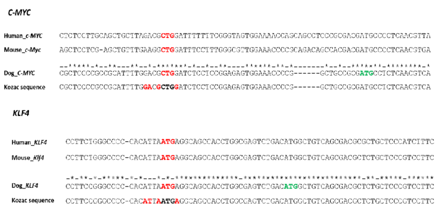

B

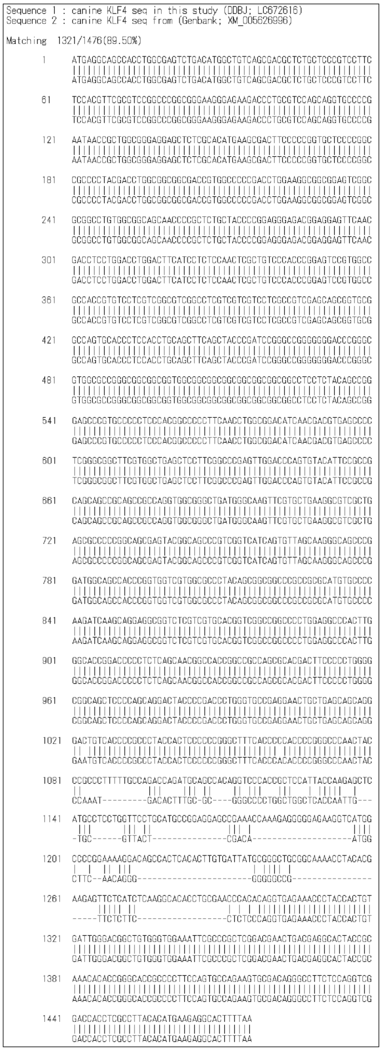

C

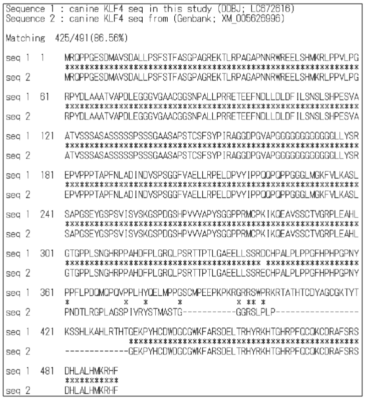

D

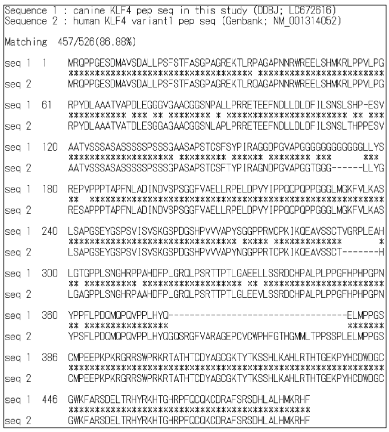

E

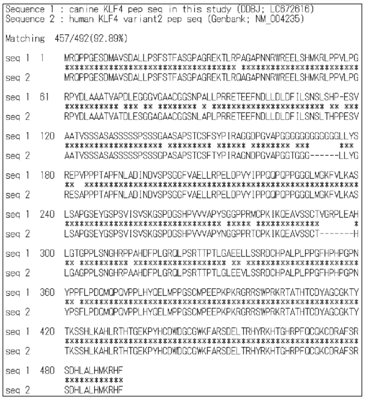

F

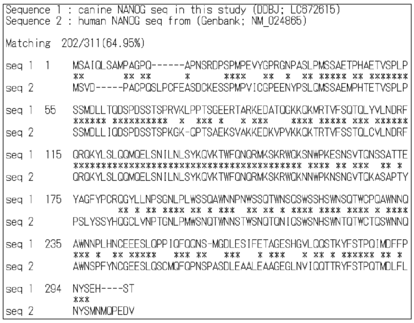

G

Gene	Canine	Human	Homology (%)
LIN28A	XM_849892.5	NM_024674.6	94.76
OCT3/4	XM_538830.4	NM_002701.6	90.83
SOX2	XM_005639752.4	NM_003106.4	98.14
C-MYC	NM_001003246.2	NM_002467.6	92.29
KLF4	LC672616	NM_004235.6	92.89
NANOG	LC672615	NM_024865.4	64.95

H

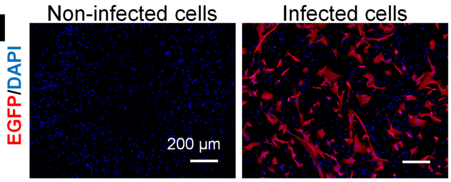

I

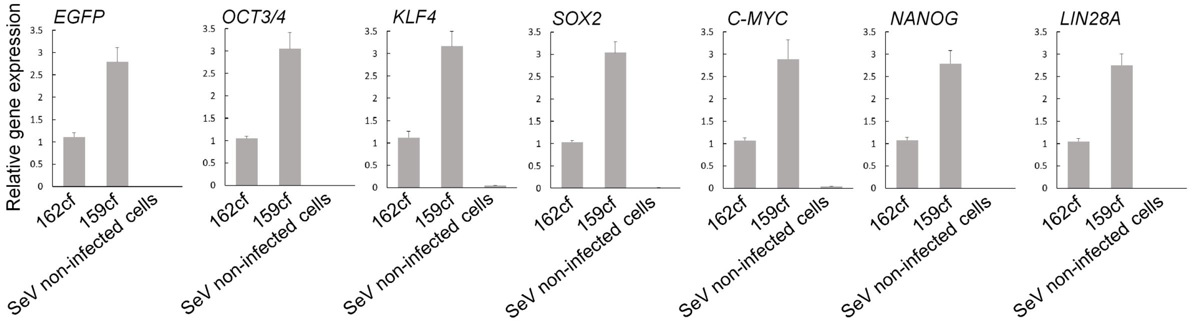

Figure S2, Reprogramming CEFs with feeder cells

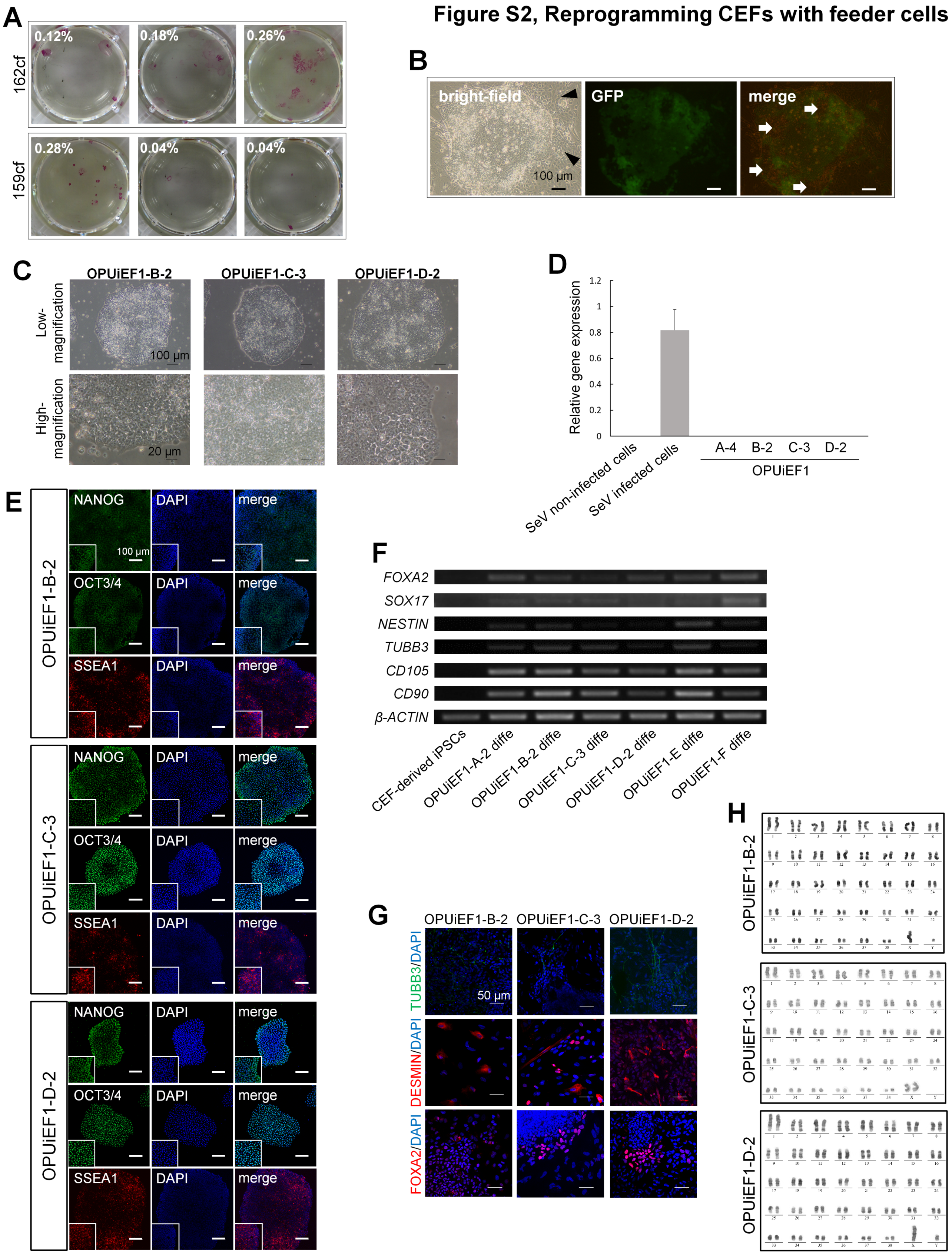

Figure S3, Reprogramming CDFs with feeder cells

A

	159cf	162cf
#1 (n = 2)	0.0393%	0
#2 (n = 1)	0.00870%	0.00870%

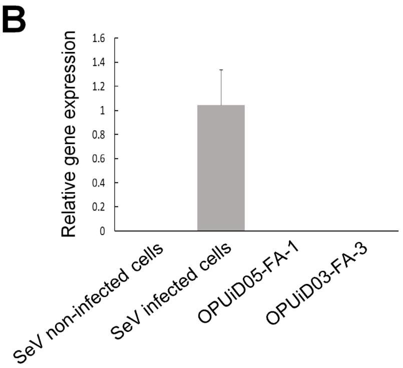

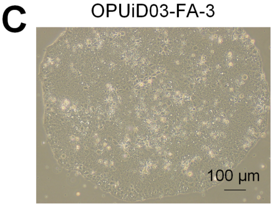

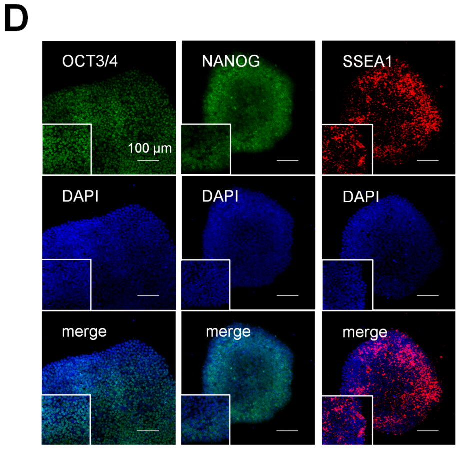

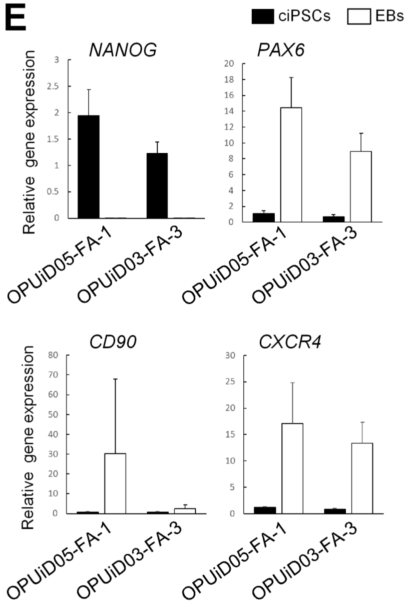

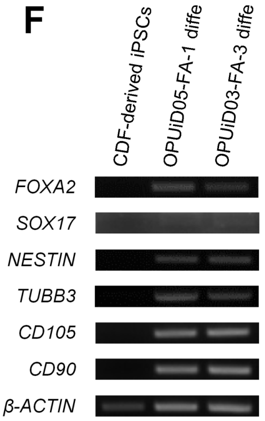

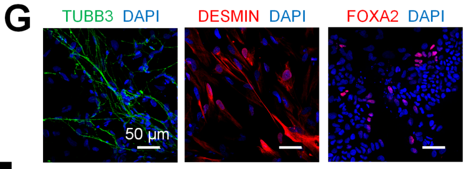

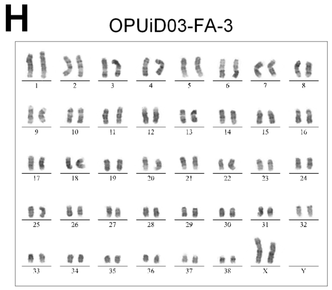

Figure S4, Comparison of cUC derivation method

A

Cell number at day 10

		isolation method	
individuals		conventional	combination
	No. 1	N/D	3.8×10^5
	No. 2	N/D	1.7×10^5
	No. 3	2.2×10^3	4.8×10^4
	No. 4	1.9×10^4	3.4×10^4
	No. 5	2.1×10^4	7.9×10^4

B

Doubling time (days)

		isolation method	
individuals		conventional	combination
	No. 1	N/D	0.51
	No. 2	N/D	0.99
	No. 3	*1	0.83
	No. 4	2.91	0.55
	No. 5	*1	1.60

C

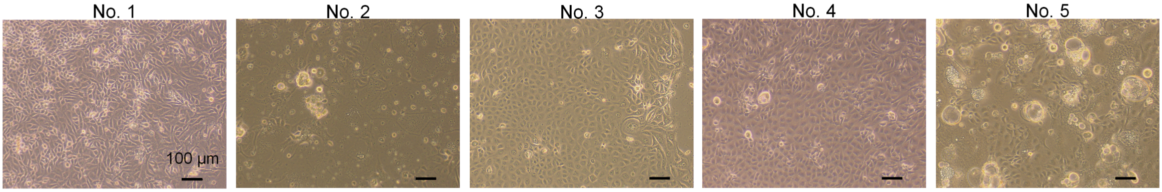

D

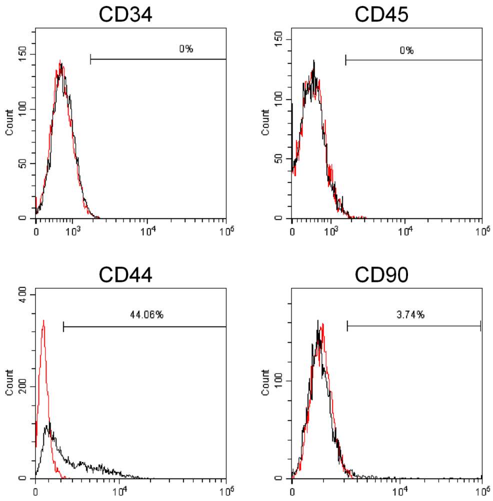

E

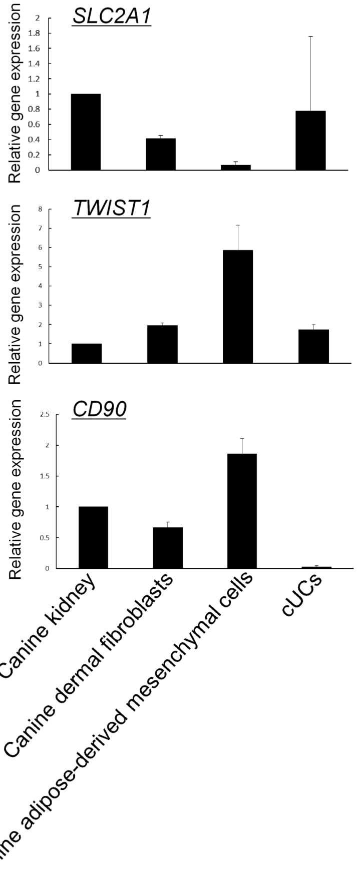

Figure S5, Reprogramming cUCs with feeder cells

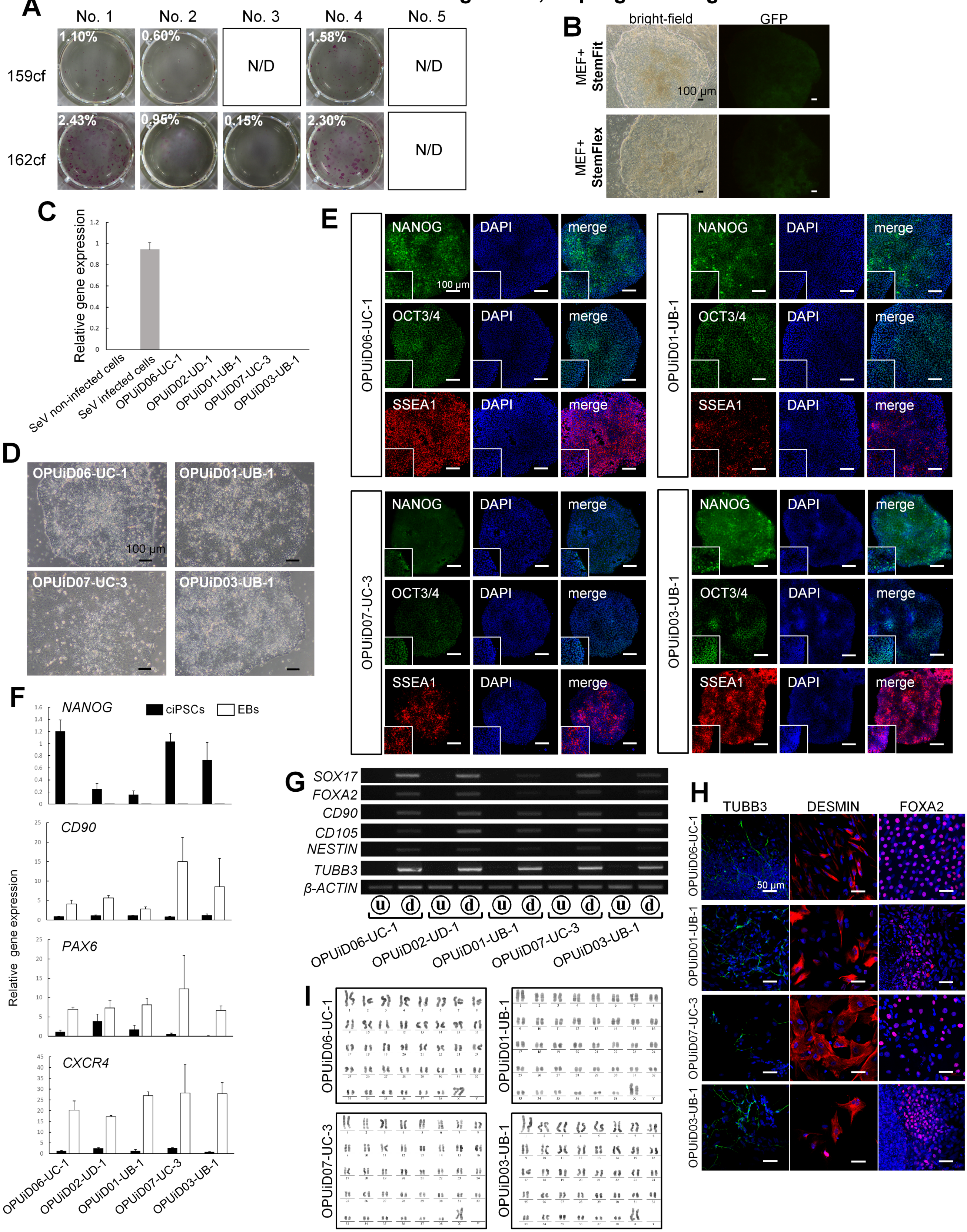

Figure S6, ciPSC generated without feeder cells

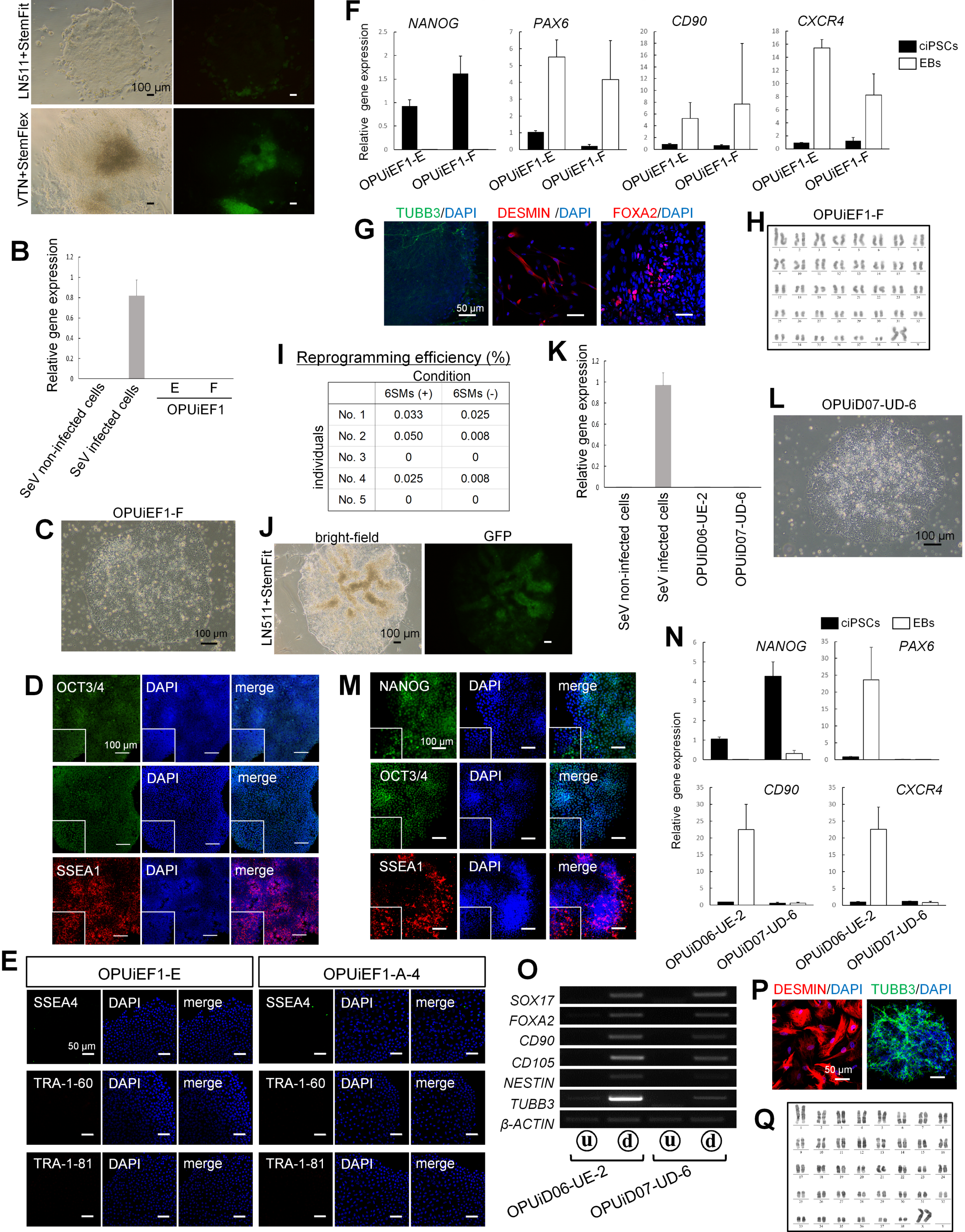

Reprogramming efficiency (%)

		Condition	
		6SMs (+)	6SMs (-)
individuals	No. 1	0.033	0.025
	No. 2	0.050	0.008
	No. 3	0	0
	No. 4	0.025	0.008
	No. 5	0	0

Supplemental Figure Legends

Figure S1 (related to Figure 1) Comparison of pluripotency associated genes and function of canine 6-factors Sendai virus (SeV)
(A) Comparison of *C-MYC* and *KLF4* sequences from GenBank. Bases in red font in human and mouse genes represent start codons. Bases in green font in dog *C-MYC* and *KLF4* are start codons from predicted data registered in GenBank. The start codon determined in this study is shown in red font and is 39 bases upstream of *C-MYC* and 27 bases upstream of *KLF4*. These start codon locations are identical to those of human and mouse genes. Kozak sequences are presented at the bottom.
(B) Sequencing results for *KLF4* (canine *KLF4* seq in this study) and sequence comparison with the sequence from XM_005626996 (canine *KLF4* seq from GenBank). The homology is 89.50%.
(C) Amino acid homology between KLF4 sequence determined in this study (canine KLF4 pep seq in this study) and canine KLF4 sequence from GenBank (canine KLF4 pep seq from GenBank). The homology is 86.56%.
(D) Comparison between canine KLF4 determined in this study and human KLF4 splicing variant 1. The homology is 86.88%.
(E) Comparison between canine KLF4 determined in this study and human KLF4 splicing variant 2. The homology is 92.89%.
(F) Comparison between canine NANOG determined in this study and human NANOG. The homology is 64.95%.
(G) Summary of homology for six reprogramming genes between canine and human.
(H) Immunostaining of enhanced green fluorescence protein (EGFP). Infection efficiency was calculated from EGFP-positive cells and total cells. Infection efficiency was 33.7% (n=3).
(I) qPCR analysis of canine pluripotency-associated genes and *EGFP* in 162cf or 159cf-infected canine embryonic fibroblasts (CEFs) and Sendai virus (SeV) non-infected cells. *β-ACTIN* was used as a normalization control gene. The relative gene expression levels were determined relative to expression in 162cf-infected cells.

Figure S2 (related to Figure 1 and 2) Reprogramming of CEFs using canine six-factors SeV.
(A) Alkaline phosphatase staining of primary colonies. The reprogramming efficiency of each event is shown.
(B) Images obtained after one small interfering RNA (siRNA) procedure. The black arrowhead shows differentiated cells, and the white arrow shows EGFP-negative undifferentiated cells. Scale bar = 100 µm.
(C) Morphologies of each ciPSCs, OPUiEF1-B-2, -C-3, and -D-2. Scale bar = 100 or 20 µm.
(D) qPCR for SeV vector in four CEF-derived iPSCs. SeV non-infected cells and SeV-infected cells were used as negative and positive control, respectively. *β-ACTIN* was used as a normalization control gene.
(E) Immunocytochemistry of OPUiEF1-B-2, OPUiEF1-C-3, and OPUiEF1-D-2 for pluripotent markers OCT3/4, NANOG, and SSEA1. Scale bar = 100 µm. High-magnification images are shown as insets.
(F) RT-PCR of differentiation markers after spontaneous differentiation of CEF-derived iPSCs under two-dimensional conditions. Endodermal markers *FOXA2* and *SOX17*, ectodermal markers *NESTIN* and *TUBB3*, and mesodermal markers *CD105* and *CD90*. *β-ACTIN* was used as a normalization control gene; CEF-derived iPSCs were used as negative controls.
(G) Immunocytochemistry of each differentiation marker after spontaneous differentiation. Ectodermal marker TUBB3, mesodermal marker DESMIN, and endodermal markers FOXA2. Scale bar = 50 µm.
(H) Karyotype analysis of each CEF-derived iPSCs, OPUiEF1-B-2, OPUiEF1-C-3, and OPUiEF1-D-2.

Figure S3 (related to Figure 3) Reprogramming of canine dermal fibroblasts (CDFs) using canine six-factors SeV and characteristics of CDF-derived iPSCs
(A) Reprogramming efficiency of CDFs from two individuals.
(B) qPCR of SeV vector for two CDF-derived iPSCs. SeV non-infected cells and SeV infected cells were used as negative and positive control, respectively. *β-ACTIN* was used as a

normalization control gene.
(C) Morphologies of OPUiD03-FA-3. Scale bar = 100 μm.
(D) Immunocytochemistry of OPUiD03-FA-3 for pluripotent markers OCT3/4, NANOG, and SSEA1. Scale bar = 100 μm.
(E) qPCR analysis of undifferentiated and differentiation markers 12 days after embryoid body (EB) formation. Black bar and white bar represent ciPSCs and EBs, respectively. Undifferentiated marker *NANOG*, ectodermal marker *PAX6*, mesodermal marker *CD90*, and endodermal marker *CXCR4*. *β-ACTIN* was used as a normalization control gene, and data are shown as the mean ± standard deviation (n = 3).
(F) RT-PCR for differentiation markers after spontaneous differentiation of CDF-derived iPSCs under two-dimensional conditions. Endodermal markers *FOXA2* and *SOX17*, ectodermal markers *NESTIN* and *TUBB3*, and mesodermal markers *CD105* and *CD90*. *β-ACTIN* was used as a normalization control gene; CDF-derived iPSCs were used as negative controls.
(G) Immunocytochemistry of each differentiation marker of OPUiD03-FA-3 after spontaneous differentiation via EB formation. Ectodermal marker TUBB3, mesodermal marker DESMIN, and endodermal markers FOXA2. Scale bar = 50 μm.
(H) Karyotype analysis of OPUiD03-FA-3 at passage 15.

Figure S4 (related to Figure 4) Comparison of two isolation methods of canine urine-derived cells (cUCs).
(A) Cell number at day10 between two isolation methods. N/D in dog No. 1 and No. 2 means not performed because urine sample was not enough to divide into two groups.
(B) Doubling time of cUCs isolated using each isolation method. N/D in dog No. 1 and No. 2 means not performed. *1 in dog No. 3 and No. 5 means that cell growth was not observed after culture.
(C) Morphologies of cUCs from each individual. Scale bar = 100 μm.
(D) Flow cytometry analysis of cUCs for CD34, CD44, CD45, and CD90. Isotype control for each marker was used as negative control and is indicated by a red line.
(E) qPCR for *SLC2A1* (renal epithelial marker), *TWIST1* and *CD90* (mesenchymal marker). Expression levels in each sample were standardized to the level of *β-ACTIN*. The data are presented as the mean ± standard deviation (n = 4), except for canine kidney data, which represents a single repricate and is included as supplemental reference data.

Figure S5 (related to Figure 4) Reprogramming of cUCs and characteristics of cUC-derived iPSCs.
(A) Alkaline phosphatase staining of primary colonies obtained from each condition. N/D in dogs No. 3 and No. 5 indicates no observation of primary colonies. The right table shows the reprogramming efficiencies. Each reprograming experiment was performed one time.
(B) Primary colony morphologies generated under StemFit and StemFlex. Scale bar = 100 μm.
(C) qPCR of SeV vector for five cUC-derived iPSCs. SeV non-infected cells and SeV infected cells were used as negative and positive control, respectively. *β-ACTIN* was used as a normalization control gene.
(D) Morphologies of ciPSCs, OPUiD06-UC-1, OPUiD01-UB-1, OPUiD07-UC-3, and OPUiD03-UB-1. Scale bar = 100 μm.
(E) Immunocytochemistry of cUC-derived iPSCs for pluripotent markers OCT3/4, NANOG, and SSEA1. Scale bar = 100 μm. High-magnification images are shown as insets.
(F) qPCR analysis of undifferentiated and differentiation markers at 12 days after EB formation. Black bar and white bar represent ciPSCs and EBs, respectively. Undifferentiated marker *NANOG*, ectodermal marker *PAX6*, mesodermal marker *CD90*, and endodermal marker *CXCR4*. *β-ACTIN* was used as a normalization control gene, and data are shown as the mean ± standard deviation (n = 3).
(G) RT-PCR of differentiation markers after spontaneous differentiation of cUC-derived iPSCs under two-dimensional conditions. Endodermal markers *FOXA2* and *SOX17*, ectodermal markers *NESTIN* and *TUBB3*, and mesodermal markers *CD105* and *CD90*. *β-ACTIN* was used as a normalization control gene; cUC-derived iPSCs were used as negative controls (u). The left right lanes of each ciPSC line show the cells after differentiation (d).
(H) EB formation from cUC-derived iPSCs and immunocytochemistry of EBs for each differentiation marker. Ectodermal marker TUBB3, mesodermal marker DESMIN, and

endodermal markers FOXA2. Scale bar = 50 µm.
(I) Karyotype analysis of OPUiD06-UC-1, OPUiD01-UB-1, OPUiD07-UC-3, and OPUiD03-UB-1 at passage 15, 11, 15, and 15, respectively.

Figure S6 (related to Figure 5 and 6) Characteristics of iPSCs generated under feeder-free conditions.
(A) Morphologies of primary colonies obtained from CEFs under combinations of iMatrix-511 (LN511) with StemFit or Vitronectin (VTN) with StemFlex. Scale bar = 100 µm.
(B) qPCR of SeV vector for OPUiEF1-E and -F. SeV non-infected cells and SeV-infected cells were used as negative and positive control, respectively. *β-ACTIN* was used as a normalization control gene.
(C) Morphologies of OPUiEF1-F. Scale bar = 100 µm.
(D) Immunocytochemistry of OPUiEF-F for pluripotent markers OCT3/4, NANOG, and SSEA1. Scale bar = 100 µm. High-magnification images are shown as insets.
(E) Immunocytochemistry of CEF-derived iPSCs under feeder-free conditions (OPUiEF1-E) and with feeder cells (OPUiEF1-A-4) for SSEA4, TRA-1-60, and TRA-1-81. Scale bar = 50 µm. Both ciPSCs did not express these undifferentiated markers.
(F) qPCR analysis of undifferentiated and differentiation markers 12 days after EB formation from CEF-derived iPSCs. Black bar and white bar represent ciPSCs and EBs, respectively. Undifferentiated marker *NANOG*, ectodermal marker *PAX6*, mesodermal marker *CD90*, and endodermal marker *CXCR4*. *β-ACTIN* was used as a normalization control gene, and data are shown as the mean ± standard deviation (n = 3).
(G) Immunocytochemistry for each differentiation marker of OPUiEF1-F after spontaneous differentiation under two-dimensional conditions. Ectodermal marker TUBB3, mesodermal marker DESMIN, and endodermal markers FOXA2. Scale bar = 50 µm.
(H) Karyotype analysis of OPUiEF1-F at passage 28.
(I) Reprogramming efficiencies of cUCs. Each reprograming experiment was performed one time.
(J) Primary colony morphologies generated from cUCs under the combination of iMatrix-511 (LN511) and StemFit. Scale bar = 100 µm.
(K) qPCR of SeV vector in both ciPSCs, OPUiD06-UE-2 and OPUiD07-UD-6. SeV non-infected cells and SeV-infected cells were used as negative and positive control, respectively. *β-ACTIN* was used as a normalization control gene.
(L) Morphologies of ciPSCs, OPUiD07-UD-6. Scale bar = 100 µm.
(M) Immunocytochemistry of OPUiD07-UD-6 for undifferentiated markers. Scale bar = 100 µm.
(N) qPCR analysis of undifferentiated and differentiation markers at 12 days after EB formation from cUC-derived iPSCs. Black bar and white bar represent ciPSCs and EBs, respectively. Undifferentiated marker *NANOG*, ectodermal marker *PAX6*, mesodermal marker *CD90*, and endodermal marker *CXCR4*. *β-ACTIN* was used as a normalization control gene, and data are shown as the mean ± standard deviation (n = 3). OPUiD07-UD-6 did not express differentiation markers.
(O) RT-PCR of differentiation markers after spontaneous differentiation of OPUiD06-UE-2 and OPUiD07-UD-6 under two-dimensional conditions. Endodermal markers *FOXA2* and *SOX17*, ectodermal markers *NESTIN* and *TUBB3*, and mesodermal markers *CD105* and *CD90*. *β-ACTIN* was used as a normalization control gene; iPSCs before differentiation were used as negative controls (u). Right lanes of each ciPSC line show the cells after differentiation (d).
(P) Immunocytochemistry of each differentiation marker of OPUD07-UD-6 after spontaneous differentiation via EB formation. Only mesodermal marker DESMIN and ectodermal marker TUBB3 were detected. Scale bar = 50 µm.
(Q) Karyotype analysis of OPUiD07-UD-6 at passage 19.

Table S1, Summary information on passage numbers

cell line name (OPUi)	RT-PCR for SeV	qPCR for SeV	IF (pluripotency)	qPCR (pluripotency)	IF (differentiation)	qPCR (differentiation)	RT-PCR (differentiation)	teratoma assay	karyotyping	cell source	feeder cell
EF1-A-4	5	12-14	15	12-14	10	13-16	28	10	14	CEF	yes
EF1-B-2	4	13-15	16	13-15	12	14-16	38	10	15		
EF1-C-3	12	14-17	18	14-17	27	18-20	34	17	20		
EF1-D-2	6	21-23	12	21-23	12	21-23	43	16	26		
EF1-E	11	19-21	22	19-21	19	19-21	48	21	17	CEF	no
EF1-F	11	15-17	13	15-17	21	18-20	47	30	28		
D05-FA-1	5	14-16	13	14-16	12	14-16	22	11	15	CDF	yes
D03-FA-3	3	11-13	11	11-13	9	12-14	18	8	15		
D06-UC-1	3	10-12	12	10-12	12	10-12	26	10	15	cUC	yes
D02-UD-1	4	10-12	14	10-12	14	10-12	25	12	15		
D01-UB-1	4	10-12	14	10-12	17	10-12	28	12	11		
D07-UC-3	4	15-17	13	15-17	14	15-17	23	11	15		
D03-UB-1	5	34-36	16	34-36	20	33-35	19	11	15		
D06-UE-2	11	17-19	21	17-19	20	17-19	11	12	25	cUC	no
D07-UD-6	5	10-12	17	10-12	16	10-12	9	11	19		

IF; immunofluorescence, SeV; Sendai virus, CEF; canine embryonic fibroblast, CDF; canine dermal fibroblast, cUC; canine urine derived cell

Table S2, Oligo sequence

Sequence of siRNA for SeV

	Sequence (5' → 3')
Sense	CAAUAGUUCACGCUGAAAGUG
Anti-sense	CUUUCAGCGUGAACUAUUGCU

Sequence of primers

Name		Sequence (5' → 3')
β-ACTIN	Forward	CAGCAAATGTGGATCAGC
	Reverse	CCAATCTCATCTCGGTTTC
OCT3/4	Forward	CTGAAGCAGAAGAGGATCAC
	Reverse	GCCGCAGCTTACACATATTC
NANOG	Forward	CTAGGGACCCTTCTCCAATG
	Reverse	CTTCTGTTTCTTGCCCTGG
SOX2	Forward	AACCCCAAGATGCACAACTC
	Reverse	CGGGGCCGGTATTTATAATC
KLF4	Forward	ACACTTGTGATTATGCGGGC
	Reverse	CCTCGTCAGTTCGTCCGAG
C-MYC	Forward	TCCTCCGGAGAGTGGAAACC
	Reverse	CCGAGTCGTAGTCGAGGTCA
LIN28A	Forward	AGAACATGCAGAAGCGCAGA
	Reverse	TTGCATTCCTTGGCATGGTG
EGFP	Forward	GGCACAAGCTGGAGTACAAC
	Reverse	ATGCCGTTCTTCTGCTTGTC
NESTIN	Forward	GTTCCAGAGGCTATACAGG
	Reverse	CTAGAGTGATAAGGGCTGAG
TUBB3	Forward	CCGGAACCATGGACAGCGTC
	Reverse	AGCGGAGAGAAGTAGTGACG
CD90	Forward	ACCTGGAGGAGGAAGAGGAA
	Reverse	AAAGCTCCTGGAAATGCTCA
CD105	Forward	ACCGTGGAACTCATCCAGAG
	Reverse	AGGTAAGGGTGCCAGTTGTG
FOXA2	Forward	CAAGGCCTATGAACAGGTG
	Reverse	CTGGTAGTAGGAGGTGTCTGC
SOX17	Forward	AAGATGCTGGGCAAGTCG
	Reverse	CCCTCCACCCGCTTCAG
SeV	Forward	GGAAGGAATCGGCTCAGTGATG
	Reverse	GGGCCGTGTTCATGGTCAC
SLC2A1	Forward	GATCGGCTCCTTCTCTGTGG
	Reverse	AGGACTTGGCCAGTTTCGAG
TWIST1	Forward	AGCTACGCCTTCTCGGTCTG
	Reverse	CTGTCCATTTTCTCCTTCTCTGG
CDH2	Forward	AGCACCCTCCTCAGTCAACG
	Reverse	TGTCAACATGGTCCCAGCA
ZEB1	Forward	GTGTGGGGTGTGAGAACTTGA
	Reverse	TCTGGTTCTCTTGAAAGGTTAGG
EPCAM	Forward	TCGCTGTCATTGTGGTTGTG
	Reverse	TGCATCTCACCCATCTCCTTT
CDH1	Forward	TCCTGGGCAGGGTGAGTT
	Reverse	GAGGCCGCTTGACTGTAATC
UTF1	Forward	GCTGCTGACCCTGAACCAG
	Reverse	CCCAAGATGAAGCCCACGG
ESRRB	Forward	GGAGGCGTGCTAGAGATGAA
	Reverse	GGACTGGTCACCACTAAGGG
GATA4	Forward	ACCAGCAGCAGTGAAGAGATG
	Reverse	AGATGTGTAGCCTTGTGGGG
GATA6	Forward	CACTACTTGTGCAACGCCTG
	Reverse	CACAAGACAATCCAAGCCGC
PAX6	Forward	TGCTGGACAATCAAAACGTGTC
	Reverse	TGGAGTCGCTACTCTCGGTT
CXCR4	Forward	GTTGAGGCTGTGGCAAACTG
	Reverse	GTAGACCACCTTTTCCGCCA

Table S3, Antibody

Primary antibodies for immunocytochemistry

Antigen	Cat.no.	Source	Final concentration
OCT3/4	sc-5279	Santa Cruz Biotechnology	0.4 μg/mL
NANOG	ab77095	Abcam	5 μg/mL
SSEA1	MAB4301	Millipore	1:100
TUBB3	MAB1637	Millipore	1:400
DESMIN	ab82506	Abcam	1 μg/mL
FOXA2	720061	Thermo Fisher Scientific	2.5 μg/mL
GFP	A-6455	Thermo Fisher Scientific	1:1000

Secondary antibodies for immunocytochemistry

	Cat.no.	Source	Final concentration
Goat anti-Mouse IgG, Alexa Fluor 488	A11029	Invitrogen	1 μg/mL
Goat anti-Rabbit IgG, Alexa Fluor 546	A11010	Invitrogen	4 μg/mL
Rabbit anti-Goat IgG, Alexa Fluor 488	A27012	Invitrogen	1 μg/mL
Goat anti-Mouse IgM, Cy3	AP128C	Chemicon	1 μg/mL

Antibodies for flow cytometry

Antigen	Cat.no.	Source	Final concentration
CD34	12-0340-42	Thermo Fisher Scientific	2.5 μg/mL
CD44	11-5440-42	Thermo Fisher Scientific	1.25 μg/mL
CD45	11-5450-42	Thermo Fisher Scientific	2.5 μg/mL
CD90	12-5900-42	Thermo Fisher Scientific	5 μg/mL

Supplemental experimental procedures

Sequencing of canine pluripotency-associated genes

NANOG was resequenced using ENSCAFT00000022072.3 (Ensembl 94) as the genomic DNA template; each exon was resequenced. *KLF4* was resequenced using cDNA from the canine ovary as a template. KOD FX DNA polymerase (Toyobo, Osaka, Japan) and a Takara Dice Touch thermal cycler (Shiga, Japan) were used for PCR. Sequencing was outsourced to Eurofins Genetics (Tokyo, Japan).

The 5′-Full RACE Core Set (Takara) was used for 5′ rapid amplification of cDNA ends (RACE) to identify the start codon of canine *KLF4* and *C-MYC* using reverse transcribed cDNA from the canine ovary as a template. The gene-specific phosphorylated primer sequences used in the 5' RACE were KLF4: TCAGCAGTTCCTC-P and C-MYC: TGATCTGTTTCAGG-P. P were used. The sequences of the primers used for 1st and 2nd PCR were KLF4 (1st-Forward: GGAATGTACACTGGGTCCAACT, 1st-Reverse: GTCATCAGTGTTAGCAAGGG, 2nd-Forward: AGTTGGAGAGGATGAAGTCCAG, 2nd-Reverse: CAGCTCCCCAGCAGGACTAC), C-MYC (1st-Forward: TTTCTTCCAGATATCCTCGCTG, 1st-Reverse: TCAAAAACATCATCATCCAGGA, 2nd-Forward: CTGCTGGTAGAAGTTCTCCTCC, 2nd-Reverse: GGAAGAACAAGGACGAAGAA).

Construction of canine six factors-SeV

Canine six factors-SeV was constructed according to the method described in the patent[1]. Briefly, full-length genome cDNA installed with the six canine pluripotency-associated genes (Oct3/4, Klf2, Sox2, c-Myc, Nanog and Lin28), the codon-optimized Sendai virus NP, P, C and L genes, the enhanced green fluorescent protein (EGFP) gene and puromycin N-acetyl transferase (PuroR) gene was constructed on a low-copy plasmid vector with P15A replication origin. This cDNA and the plasmid vectors for expressing Sendai virus NP, P and L proteins were transfected to BHK-21 cells expressing T7 RNA polymerase for reconstituting active nucleocapsid in the cell. Finally, the plasmid vectors for expressing Sendai virus F, HN and M proteins were transfected to the BHK-21 cells containing the vector genome for producing Canine six factors-SeV. SeV 159cf vector and SeV 162cf vector have different gene expression levels. 159cf expresses the installed genes about three times higher than 162cf does, by controlling NP gene expression[1].

Composition of culture media

Feeder medium is composed of Dulbecco’s modified Eagle’s medium with high glucose (DMEM high-glucose; Nacalai Tesque, Kyoto, Japan) containing 10% fetal bovine serum (FBS; Kibbutz Beik Haemek, Israel), 2 mM L-glutamine (Nacalai Tesque), 100 U/mL penicillin, and 100 µg/mL streptomycin (Nacalai Tesque). The primary medium was composed of DMEM high glucose and DMEM/Nutrient Mixture F-12 Ham (Nacalai Tesque) mix (1:1) supplemented with 10% FBS, 100 U/mL penicillin, 100 µg/mL streptomycin, 2.5 µg/mL amphotericin B, and REGM SingleQuot kit supplements (Lonza, Basel, Switzerland). RE/MC proliferation medium was mixed RE proliferation medium and MC proliferation medium (1:1). RE proliferation medium is RE cell basal medium containing REGM Bullet Kit (Lonza). MC proliferation medium was DMEM high-glucose supplemented with 10% FBS, 100 U/mL penicillin, 100 µg/mL streptomycin, GlutaMAX (1×, Thermo Fisher Scientific, Waltham, MA, USA), 0.1 mM minimal essential medium nonessential amino acids (MEM NEAA; Thermo Fisher Scientific), 5 ng/mL basic fibroblast growth factor (Thermo Fisher Scientific), 5 ng/mL platelet derived growth factor-AB (Peprotech, Rocky Hill, NJ, USA), and 5 ng/mL epidermal growth factor (Thermo Fisher Scientific). N2B27 medium consisted of DMEM/Nutrient Mixture F-12 Ham supplemented with N2 supplement (1×, Thermo Fisher Scientific), B27 supplement (1×, Thermo Fisher Scientific), GlutaMAX, 0.1 mM MEM NEAA, and 0.1 mM 2-mercaptoethanol (Sigma Aldrich, St. Louis, MO, USA). The 20% FBS medium consisted of DMEM/Nutrient Mixture F-12 Ham supplemented with 20% FBS (Sigma Aldrich), 2mM L-glutamine, 100 U/mL penicillin, 100 µg/mL streptomycin, 0.1 mM MEM NEAA, and 0.1 mM 2-mercaptoethanol. The small molecule cocktails added to N2B27 medium were composed of 10 µM Y-27632 (Nacalai Tesque), 0.5 µM PD0325901 (Reprocell, Kanagawa, Japan), 3 µM CHIR99021 (Fujifilm Wako Pure Chemical Corporation, Osaka, Japan), 0.5 µM A83-01 (Stemgent, San Diego, CA, USA), named “4SMs,” or 10 µM forskolin (Tocris, Bristol, Avon, UK), and 50 µg/mL L-ascorbic acid (Sigma Aldrich) in addition to 4SMs, named as “6SMs.”

Preparation and culture of feeder cells and canine cells

MEFs were isolated from the fetuses of ICR mice (Japan SLC, Shizuoka, Japan). CEFs were isolated from the intrauterine fetus of a beagle dog after 30 days of pregnancy. The head, visceral tissues, and liver were removed from the fetus. The remaining tissues were cut into small pieces and cultured in a feeder medium. CDFs were derived from punch biopsy tissue taken from the dorsal skin of 8- and 12-year-old beagle bitches. After chopping, the tissues were cultured in a feeder medium.

cUCs were isolated from urine samples from five dogs (from 9 to 14 years old bitches). Urine samples were obtained with a sterile catheter. cUCs were isolated as described previously[2]. Briefly, urine samples were centrifuged at 400 × g for 10 min, and the supernatant was removed. The pellet was resuspended with washing buffer, which was PBS (-) containing 100 U/mL penicillin, 100 μg/mL streptomycin, and 500 ng/mL amphotericin B (Sigma Aldrich), and centrifuged at 200 × *g* for 10 min. After removing the supernatant, for the conventional method, the pellet was resuspended in 1 mL of primary medium, seeded into a gelatin-coated 12-well dish, and incubated at 37°C and 5% CO_2 (day 0). After 24, 48, and 72 h, 1 mL primary medium was added to the 12-well dish. On day 4, the primary medium was removed leaving 1 mL, and 1 mL RE/MC proliferation medium was added. From day 5, half of the medium was replaced every day. For the combination method, the pellet was resuspended in 1 mL of primary medium containing 10 μM Y-27632 (Nacalai Tesque) and seeded onto Matrigel (Corning, Inc., Corning, New York, NY) coated (1:25) 12-well dish. The medium was changed in the same manner as in the conventional method by adding 10 μM Y-27632.

MEFs, CEFs, CDFs, and cUCs were passaged using 0.25% trypsin-EDTA (Sigma Aldrich) and stored at -80°C using a cell banker (Nippon Zenyaku Kogyo, Fukushima, Japan).

Reprogramming canine cells without feeder cells

When we reprogrammed canine cells with other feeder-free culture system, we altered DEF-CS to other feeder-free culture system, iMatrix-511 with StemFit or Vitronectin (VTN-N) Recombinant Human Protein, Truncated (Vitronectin; Thermo Fisher Scientific) with StemFlex (Thermo Fisher Scientific). Primary colonies were passaged onto iMatrix-511-coated dishes and maintained in StemFit.

ciPSC cryopreservation

ciPSCs were dissociated with TrypLE Select (Thermo Fisher Scientific) and stored at −80°C or −276°C using a stem cell banker (Nippon Zenyaku Kogyo, Fukushima, Japan).

RT-PCR and quantitative RT-PCR

Total RNA was extracted using the RNeasy Micro Kit (Qiagen, Hilden, Germany). Reverse transcription (RT) was performed using random primers and ReverTra Ace (Toyobo). Polymerase chain reaction (PCR) was performed using a Blend Taq Plus (Toyobo). PCR products were resolved on a 2% agarose gel stained with ethidium bromide and observed using an ultraviolet transilluminator (AE-9020; ATTO, Tokyo, Japan).

To quantify mRNA expression levels, PCR was performed using PowerTrack™ SYBR™ Green Master Mix (Thermo Fisher Scientific) and a QuantStudio 12K Flex real-time PCR machine (Theremo Fisher Scientific). *β-ACTIN* was used as a normalization control gene, and relative gene expression levels were calculated by ΔΔCt method[3]. All primers are listed in Table S2.

Immunocytochemistry

The cells were fixed in 4% paraformaldehyde, permeabilized with 0.1% Tween 20 in PBS (-) and blocked with 10% bovine serum albumin (Fujifilm Wako Pure Chemical Corporation). The cells were then incubated with primary antibodies at 4°C overnight. Negative control cells were incubated in PBS without primary antibodies. On the next day, the cells were washed, incubated with appropriate secondary antibodies at 20–25°C for 1 h, and mounted using ProLong Gold Antifade Reagent with 4′,6-diamidino-2-phenylindole (Thermo Fisher Scientific) to label DNA. Immunolabeled cells were observed using a confocal laser microscope (FV3000; Olympus, Tokyo, Japan). All antibodies are listed in Table S3.

Supplemental reference

1. Nakanishi, M., and Iijima, M. (2020). GENE EXPRESSION SYSTEM USING STEALTHY RNA, AND GENE INTRODUCTION/EXPRESSION VECTOR INCLUDING SAID RNA. United States Patent No. 10544431 B1.

2. Xu, Y., Zhang, T., Chen, Y., Shi, Q., Li, M., Qin, T., Hu, J., Lu, H., Liu, J., and Chen, C. (2020). Isolation and Characterization of multipotent canine urine-derived stem cells. Stem Cells Int. 2020, 8894449 10.1155/2020/8894449.

3. Livak, K.J., and Schmittgen, T.D. (2001). Analysis of relative gene expression data using real-time quantitatiive PCR and the 2−ΔΔCT method. Methods 25, 402–408. 10.1006/meth.2001.1262.
